# Down-regulation of hepatic expression of GHR/STAT5/IGF-1 signaling pathway fosters development and aggressiveness of HCV-related hepatocellular carcinoma: Crosstalk with Snail-1 and type 2 transforming growth factor-beta receptor

**DOI:** 10.1371/journal.pone.0277266

**Published:** 2022-11-14

**Authors:** Mona A. Abu El-Makarem, Mariana F. Kamel, Ahmed A. Mohamed, Hisham A. Ali, Mahmoud R. Mohamed, Alaa El-Deen M. Mohamed, Ahmed M. El-Said, Mahmoud G. Ameen, Alshymaa A. Hassnine, Hatem A. Hassan

**Affiliations:** 1 Department of Internal Medicine, School of Medicine, Minia University, Minia, Egypt; 2 Department of Pathology, School of Medicine, Minia University, Minia, Egypt; 3 Department of Pathology, Minia Oncology Center, Minia, Egypt; 4 Department of Pathology, South Egypt Cancer Institute, Assuit University, Assuit, Egypt; 5 Department of Tropical Medicine and Gastroenterology, School of Medicine, Minia University, Minia, Egypt; Texas A&M University, UNITED STATES

## Abstract

**Background and aims:**

So far, few clinical trials are available concerning the role of growth hormone receptor (GHR)/signal transducer and activator of transcription 5 (STAT5)/insulin like growth factor-1 (IGF-1) axis in hepatocarcinogenesis. The aim of this study was to evaluate the hepatic expression of GHR/STAT5/IGF-1 signaling pathway in hepatocellular carcinoma (HCC) patients and to correlate the results with the clinico-pathological features and disease outcome. The interaction between this signaling pathway and some inducers of epithelial-mesenchymal transition (EMT), namely Snail-1 and type 2 transforming growth factor-beta receptor (TGFBR2) was studied too.

**Material and methods:**

A total of 40 patients with HCV-associated HCC were included in this study. They were compared to 40 patients with HCV-related cirrhosis without HCC, and 20 healthy controls. The hepatic expression of GHR, STAT5, IGF-1, Snail-1 and TGFBR2 proteins were assessed by immunohistochemistry.

**Results:**

Compared with cirrhotic patients without HCC and healthy controls, cirrhotic patients with HCC had significantly lower hepatic expression of GHR, STAT5, and IGF-1proteins. They also displayed significantly lower hepatic expression of TGFBR2, but higher expression of Snail-1 versus the non-HCC cirrhotic patients and controls. Serum levels of alpha-fetoprotein (AFP) showed significant negative correlations with hepatic expression of GHR (r = -0.31; p = 0.029) and STAT5 (r = -0.29; p = 0.04). Hepatic expression of Snail-1 also showed negative correlations with GHR, STAT5, and IGF-1 expression (r = -0.55, p = 0.02; r = -0.472, p = 0.035, and r = -0.51, p = 0.009, respectively), whereas, hepatic expression of TGFBR2 was correlated positively with the expression of all these proteins (r = 0.47, p = 0.034; 0.49, p = 0.023, and r = 0.57, p<0.001, respectively). Moreover, we reported that decreased expression of GHR was significantly associated with serum AFP level>100 ng/ml (p = 0.048), increased tumor size (p = 0.02), vascular invasion (p = 0.002), and advanced pathological stage (p = 0.01). Similar significant associations were found between down-regulation of STAT5 expression and AFP level > 100 ng/ml (p = 0.006), vascular invasion (p = 0.009), and advanced tumor stage (p = 0.007). Also, attenuated expression of IGF-1 showed a significant association with vascular invasion (p < 0.001). Intriguingly, we detected that lower expression of GHR, STAT5 and IGF-1 were considered independent predictors for worse outcome in HCC.

**Conclusion:**

Decreased expression of GHR/STAT5/IGF-1 signaling pathway may have a role in development, aggressiveness, and worse outcome of HCV-associated HCC irrespective of the liver functional status. Snail-1 and TGFBR2 as inducers of EMT may be key players. However, large prospective multicenter studies are needed to validate these results.

## Introduction

Hepatocellular carcinoma (HCC) is a global health problem, as it is the sixth most prevalent cancer and the fourth cause of cancer-associated deaths [1]. It develops in 80–90% of patients on a background of liver cirrhosis, irrespective of the underlying etiology [2]. Among the different etiological factors of liver cirrhosis, chronic hepatitis C virus (HCV) infection contributes to the most frequent risk factor for developing HCC in Egypt [3] due to its widespread presence in this locality [4].

HCC is highly heterogeneous; from clinical point of view, ~ 80% of patients are diagnosed with advanced stage which makes palliative therapy requisite, in addition to the diverse etiological risk factors [5], and the high 5-year recurrence rate [6]. As yet, the imaging modalities used for HCC diagnosis and staging are comparatively imprecise [7], moreover, the various histopathological subtypes might contradict accurate diagnosis [8]. With respect to the molecular mechanisms, HCC is associated with multiple genetic and epigenetic alterations that co-operate with the tumor microenvironment to hasten hepatocarcinogenesis, tumor progression and metastasis [9]. As a result of these alterations, several signaling pathways are dysregulated, including growth factors e.g insulin like growth factor (IGF) [10].

The cytokine growth hormone (GH) is secreated in a pulsatile manner by somatotropic cells in the lateral wings of the anterior pituitary gland. It fulfills its effects by direct or indirect means; the direct effect is mediated by binding of GH to its receptors (GHR) on target cells by activating the mitogen activated protein kinase/extracellular signal-regulated kinase pathway, whereas the indirect effect is exerted by the way of its effectors, mainly IGF-1. While the GH is the major regulator of IGF-1 production by liver cells, IGF-1 counteracts GH synthesis by a negative feedback mechanism. IGF-1 is also synthesized by all target tissues, so it acts as an endocrine and autocrine / paracrine hormone [11].

IGF system includes 2 specific ligands; IGF-1 and IGF-2. Both IGFs play a crucial role in somatic growth and anabolic roles in various tissues and organs. IGF-1 exerts its growth promoting effects of GH during postnatal life, however, IGF-2 is responsible for prenatal and fetal growth, that is GH-independent [12]. IGFs mediate their effects through a group of cell surface receptors including type I IGF receptor (IGF-IR), type II IGF receptor (IGF-2R), insulin receptor (IR), and hybrid receptor (IGF-IR/IR). IGF-IR binds IGF-1 with higher affinity than IGF-2 and insulin, while IGF-2 is the only ligand of IGF-2R [13]. In biological fluids, levels of IGF are adjusted by a group of six IGF binding proteins (IGFBPs) that subjected to proteolysis by IGFBP- specific proteases to release bound IGF by decreasing their affinity. The majority of IGF is bound to IGFBP-3, with acid-labile subunit (ALS) forming ternary complexes that act as transporters of IGF and prolong their half-lives in the circulation [14].

GH/GHR interaction evokes activation of various tyrosine kinases including Janus Kinase 2 (JAK2) protein. Subsequently, the activated GHR-JAK 2 complex triggers phosphorylation of signal transducer and activator of transcription 5A (STAT5A) and STAT5B (both referred to as STAT5), in addition to STAT1 and STAT 3, when STAT 5 expression is low or absent. Activated STAT proteins translocate into the nucleus to initiate different gene expression. STAT5B is aminly expressed in liver cells. Hepatic STAT5B guides the transcription of IGF-1, ALS, suppressors of cytokine signaling 2, components of the cytochrome p 450 detoxifying system, as well as many genes related to glucose and lipid metabolism [15].

Taken into account the potent mitogenic and antiapoptotic role of GH and IGF-1 in all cells of the organism [16], it is not surprising that both can influence cancer risk. Compelling evidences obtained from different types of studies have demonstrated significant associations between raised serum levels of IGF-1 and increased risk of various solid tumors [17–19], including HCC [20–22]. Likewise, epidemiological studies have revealed an increased risk of a variety of cancers in patients with acromegaly [23]. Additionally, a high incidence of colorectal cancer and Hodgkin’s lymphoma has also been reported in children treated with pituitary–derived GH [24]. However, the causal role of GH and IGF-1 in oncogenesis is still debated; they might serve as permissible agents [25]. Although the activation of STAT5 system has been considered a risk factor for different human cancers [26–28], its role in HCC is context–dependent as it can act as a tumor promoter and a tumor suppressor [29]. To the best of our knowledge, the clinical studies in this research area have been relatively scarce and had discordant results.

EMT is a developmental regulatory reversible process initiated in cancer cells by which epithelial cells acquire the capability to proliferate, invade, and resist apoptosis. EMT is characterized by down-regulation of epithelial markers such as E-cadherin, Claudin family and specific cytokeratin intermediate filament proteins, combined with up-regulation of mesenchymal markers e,g., N-cadherin, α-smooth muscle actin and vimentin [30]. It is triggered by repression of E-cadherin expression via transcription factors; including zinc finger proteins, (Snail 1/2/3 and Zeb 1/2), and basic helix loop-helix proteins (Twist 1/2) [31]. E-cadherin is a cell membrane protein that binds to β-catenin; one of the components of Wnt signaling pathway which allows epithelial cells to firmly attached. Therefore, reduced expression of E-cadherin resulting in translocation of β-catenin into the nucleus leading to induction of EMT via liberation of many transcription factors [32]. At the metastatic site, the epithelial cells return toward its ancestral condition by a process known as mesenchymal-epithelial transition to return the proliferative state to produce metastatic nodules [33].

Snail-1 is the most important transcription factor responsible for E-cadherin repression by mediating histone modification [34]. Additionally, Snail-1 plays a critical role in cell survival [35], immune regulation [36] as well as, preservation of cancer stem cell-like properties [37]. Snail expression is under control of many signaling molecules released from tumor microenvironment (TME) such as epidermal growth factor, fibroblast growth factor, transforming growth factor-beta (TGF-β), Notch, Wnt, tumor necrosis factor-α, and cytokines [38].

TGF-β is a multifunctional cytokine that signals through heteromerics of type 1 and type 2 TGF-β receptors (TGFBR1, TGFBR2) which activate either Smad family via Smad 2/3/4 complexes or non-Smad cascades such as PI3K/Akt, p38MAPK, MAPK-ERK and JNK pathways [39]. Activated TGF-β could be tumor suppressive or oncogenic as determined by the context. In tumor cells, the TGF-β signaling is unregulated by various mutations or epigenetic changes, thereby; cells become resistant to the suppressive sequel of TGF-β signaling pathway [40]. Down-expression or mutations of TGFBR2 has been reported in various cancers [41–43], inclusive of HCC [44], however, the underlying mechanism has not been clarified yet.

The purpose of this study was to investigate the tissue expression of GHR/STAT5/IGF-1 signaling pathway by immunohistochemistry (IHC) in HCV–associated HCC patients and to correlate the results with the clinico-pathological features and disease outcome. The interplay between this signaling pathway and both Snail-1 and TGFBR2 as inducers of EMT, was evaluated as well.

## Subjects and methods

### Eligible subjects

The current retrospective, cross-sectional, comparative study was carried out in the Internal Medicine and Pathology Departments at Minia University Hospital, Egypt, in collaboration with the Pathology Department at Minia Oncology Center, Egypt between May 2019 and February 2021. To obtain a power of 99%, a sample size of 40 patients with HCV-related HCC was enrolled in this study. It was calculated at the level of 0.05 significance using G Power 3.19.2 Software. The study was conducted on formalin-fixed paraffin-embedded liver tissues from 40 patients with HCC on a background of HCV-associated liver cirrhosis. This group of patients was compared to two other groups: a group of HCV-related liver cirrhosis patients without HCC, and healthy controls. Data of HCC and cirrhosis patients were retrieved from their medical files in Minia Oncology Center archives. Only patients with adequate liver tissue and complete clinico-pathological data were eligible. The exclusion criteria included causes of chronic liver diseases other than chronic HCV infection, endocrinal diseases that may influence the level of GH-IGF-1 axis, diabetes mellitus, end-organ failure, organ transplantation, hepatic resection, prior locoregional treatment for HCC, extrahepatic and hematological malignancies, autoimmune diseases, as well as steroid and immunosuppressive medications.

### Hepatocellular carcinoma patients (group I)

This group included 40 consecutive patients with HCC, of whom 32(80%) were males. They were recruited from attendants of Minia Oncology Center for liver biopsy. The diagnosis of HCC was based on the characteristic imaging criteria as defined by Bruix and Sherman [45] and histological evaluation [46].

### Liver cirrhosis patients (group II)

This group comprised 40 patients with HCV-related cirrhosis (30(75%) males, and 10(25%) females). They were consecutively enrolled from those referred by outpatient clinics. The diagnosis of liver cirrhosis was built on the standard clinical criteria, in addition to the histopathological examination [47]. Presence of anti-HCV and detection of serum HCV-RNA for 6 months or more, were characteristic features of chronic HCV infection.

### Healthy controls (group III)

A total of 20 healthy subjects were prospectively collected from subjects who underwent abdominal surgery in the Department of General Surgery at Minia University Hospital. They were 15 (75%) males and 5(25%) females. All were clinically free and showed nothing abnormal in laboratory analyses.

### Informed consent

This study protocol was approved by the Institutional Ethics Committee of the School of Medicine, Minia University, and by Institutional Review Board of Minia Oncology Center, Egypt. The study was performed according to the guidelines and regulations of the 1975 Helsinki Declaration and International Conference on Harmonization Guidelines for Good Clinical Practice. Informed written consent was obtained from all subjects.

### Clinical and laboratory assessment

Demographic, clinical data, and laboratory findings including; the peripheral hemogram, liver and kidney function tests, fasting and postprandial serum glucose levels, plasma levels of alpha fetoprotein (AFP), and virological assays, as well as the imaging studies were obtained by reviewing the medical files. The functional status of the liver was evaluated by the Child-Pugh [48] and the Model for End-Stage Liver Disease (MELD) [49] scoring systems. The clinicopathological features were assessed according to the Tumor-Node-Metastasis (TNM) [50] and Okuda [51] staging systems. Concerning the healthy volunteers, they were asked to complete a questionnaire on the age, sex, tobacco and alcohol exposure, and current history of any medical illness including diabetes mellitus. They gave venous blood samples to assess the aforementioned laboratory tests using the commercially available kits according to the manufacturer’s guidance.

### Immunohistochemistry (IHC)

IHC was performed on 4-μm tissue sections taken from 10% buffered formalin-fixed, paraffin-embedded tissue blocks. Sections were deparaffinized in xylene bath and rehydrated by descending dilutions of ethyl alcohol. Hydrogen peroxide was used to block endogenous peroxidase activity. Antigen retrieval was carried out utilizing citrate buffer concentrate (pH 6). Mouse GHR monoclonal antibody (1/100 dilution, Santa Cruz Biotechnology, Texas, USA), rabbit STAT5 monoclonal antibody (1/100 dilution, Abcam Cambridge Biomedical Campus, UK), mouse IGF-1monoclonal antibody (1/50 dilution, MyBioSource, San Diego USA), mouse Snail-1 monoclonal antibody (1/50 dulation, Santa Cruz Biotechnology Texas, USA), and mouse TGFBR2 monoclonal antibody (1/100 dilution, Santa Cruz Biotechnology, Texas, USA) were used overnight as primary antibodies. Visualization was performed by Avidin-Biotin detection system (DAKO). Positive controls were used to assess correct tissue preparation and staining. One positive control tissue section for each antibody was processed in the same manner as the patient tissue samples and was included in each staining run. One negative control slide was processed for each case by omitting the primary antibody from the staining procedure. Absence of specific staining in the negative control sections was indicative of lack of secondary antibody cross-reactivity with other non-target cellular components.

#### Interpretation of GHR, STAT5 and IGF-1 immunoreactivity

The specimens were evaluated twice in different times by two experienced pathologists, blinded for the clinico-pathological data of the study subjects. The final staining scores of GHR, STAT5, IGF-1, Snail-1, and TGFBR2 were calculated by multiplying the intensity score by the percentage score. The intensity of score was regarded as: absent: 0; weak: 1, moderate: 2, and strong: 3, whereas, the percentage score was graded as follows: none: 0, 1: 1–10%, 2:11–33%, 3:34–66%, and 4: 67–100%. For statistical analysis, a final staining score ≤4 was considered as low expression and a score >4 as a high expression.

### Statistical analyses

Data were analyzed using IBM SPSS for Windows (version 20). Categorical variables were expressed as count and percentages and compared using the Chi-square test and the Fisher exact test when appropriate. One-sample Kolmogorov Smirnov test was used to test for the normality of quantitative variables. The normally distributed variables were described as mean ± standard deviation (SD). Each two groups were compared using Student’s t-test, whereas the three groups were compared using one-way analysis of variance followed by Bonferroni post-hoc test between each two groups. The non-normally distributed parameters were expressed as median and interquartile range (IQR) and compared using Kruskal Wallis test followed by Mann Whitney test between each two groups. Pearson’s correlation coefficient was used to evaluate the association between two continuous variables, while, Spearman’s correlation coefficient was used to test the relation between non-parametric variables. Overall survival (OS) was defined as the time (in months) between the date of disease diagnosis and the date of last follow up or death. Univariate and multivariate survival analysis was done by Cox proportional hazards regression model. Univariate regression models were used to detect independent factors associated with OS. Multivariate Cox analysis was conducted to evaluate meaningful variables detected by univariate analysis. Hazard ratio (HR) and its confidence interval (95% CI) were calculated for each factor. A p value ≤ 0.05 was used as a significant criterion.

## Results

The present study included forty cirrhotic patients with HCC, of whom 32 (80%) were male. The mean age at initial diagnosis was 66 ± 8.1 years. All patients were positive for anti-HCV and PCR for HCV-RNA. Among those patients, 9 (22.5%); 8 (20%), and 23 (57.5%) were class A, B, and C, respectively, according to the Child-Pugh classification. This group of patients was compared to two other groups: a group of HCV-associated cirrhosis patients without HCC and a group of healthy volunteers (c.f., Table 1).

**Table 1:** Baseline characteristics of the study groups.

Variable	HCC patients G1(n = 40)	Cirrhotic patients G2(n = 40)	Healthy controls G3(n = 20)	p-value			
				Among 3 groups	p1	p2	p3
Gender [n (%)]							
Male	32 (80%)	30 (75%)	15 (75%)	0.918	0.72*	0.72*	1*
Female	8 (20%)	10 (25%)	5 (25%)				
Age (years) [mean ± SD]	66±8.1	62.4±12.1	64.3±10.5	0.55	0.831†	1†	1†
BMI (kg/m^2^) [mean ± SD]	25.5±3.6	24±2.4	30±2.6	**<0.001**	0.259†	**<0.001†**	**<0.001†**
Smoking [n (%)]							
No	28(70%)	28(70%)	15(75%)	0.921	1*	0.72*	0.72*
Yes	12(30%)	12(30%)	5(25%)				
Platelets (1×10^3^/μl) [mean ± SD]	192±66.4	186±49.8	248.1±63	**0.003**	1†	**0.014†**	**0.006†**
Total bilirubin (mg/dl) [median(IQR)]	1.2(0.8–1.3)	1.2(0.9–1.3)	0.2(0.1–0.7)	**0.003**	0.86††	**0.01††**	**0.001††**
ALT (IU/L) [median(IQR)]	45.5(41–82)	43.5(35.5–65.3)	26(18–30)	**<0.001**	0.40††	**<0.001††**	**0.001††**
AST(IU/L) [median(IQR)]	66(39.5–110.8)	58.5(42.3–82)	28(25–32)	**<0.001**	0.39††	**<0.001††**	**<0.001††**
Serum albumin (gm/dl) [mean ± SD]	3.4±0.7	3.7±0.7	3.9±0.3	**0.04**	0.30†	**0.05†**	0.62†
PT (seconds) [mean ± SD]	14.8±2.8	13.6±2.7	11.7±1.1	**0.041**	1†	**0.04†**	0.264†
INR [mean ± SD]	1.3±0.3	1.2±0.3	1.1±0.1	**0.036**	1†	**0.03†**	0.320†
Serum creatinine (mg/dl) [mean ± SD]	0.8±0.3	0.8±0.2	0.7±0.1	0.150	1†	0.831†	0.159†
AFP (ng/dl) [median (IQR)]	86(31.8–1000)	8(5.1–341.8)	4(3–4.8)	**<0.001**	**0.01††**	**<0.001††**	**<0.001††**
Child-Pugh class [n (%)]							
A	9(22.5%)	5(12.5%)	---		0.071*	----	----
B	8(20%)	10(25%)	---				
C	23(57.5%)	25(62.5%)	----				
Child-Pugh score [mean ± SD]	9.8±2.3	9.8±2.3	----		0.60§	----	----
MELD [median (IQR)]	9.5(7–12.5)	8(7–10.8)	----		0.35††	----	----
TNM score (I+II/III+IV)[n.(%)]	24(60%)/16(40%)	----	-----		----	----	----
Okuda score (1/2+3) [n.(%)]	22(55%)/18(45%)	----	-----		----	----	----

HCC: hepatocellular carcinoma; G: group; n: number; BMI: body mass index; kg/m^2^: kilogram/meter^2^; ALT: alanine aminotransferase; AST: aspartate transaminase; PT: prothrombin time; INR: international normalized ratio; AFP: alpha-fetoprotein; MELD: model of end stage liver disease; TNM: Tumor-Node-Metastasis

*: Chi square test

†: one way analysis of variance test followed by Bonferroni post-hoc test between each two groups

††: Kruskal Wallis test followed by Mann Whitney test between each two groups

§: Student’s t-test; SD: standard deviation; IQR: interquartile range; p1: G1 vs. G2; p2: G1 vs. G3; p3: G2 vs. G3.

Bold values denote significant results

### Cirrhotic patients with HCC versus those without

Group I included 40 cirrhotic patients with HCC, whereas group II consisted of 40 cirrhotic patients without HCC. The baseline characteristics of both groups are depicted in Table 1. There was no statistically significant difference in age, sex, BMI, smoking exposure, platelet count, liver function tests, functional status of the liver, and serum creatinine. However, cirrhotic patients with HCC had significantly higher blood levels of AFP as compared to those without [86(31.8–1000)ng/ml vs. 8(5.1–341.8)ng/ml, p = 0.01]. (c.f., Table 1).

### Cirrhotic patients with HCC versus healthy volunteers

Cirrhotic patients with HCC were matched with healthy volunteers as regards age, sex, smoking exposure, and serum creatinine. The serum levels of total bilirubin, ALT, AST, PT, INR and AFP were significantly higher in HCC patients than in healthy controls [1.2(0.8–1.3)mg/dl vs.0.2(0.1–0.7) mg/dl, p = 0.01; 45.5(41–82)IU/L vs. 26(18–30)IU/L, p<0.001; 66(39.5–110.8)IU/L vs. 28(25–32)IU/L, p<0.001; 14.8±2.8sec. vs. 11.7±1.1sec., p = 0.04; 1.3±0.3 vs. 1.1±0.1, p = 0.03, and 86(31.8–1000)ng/ml vs.4(3–4.8)ng/ml, p<0.001, respectively. Whilst, they displayed statistically significant lower values of BMI, platelet count, and serum albumin versus healthy control group (25.5 ± 3.6 kg/m^2^ vs. 30 ±2.6 kg/m^2^, p<0.001; 192±66.4 (1×10^3^ μl) vs. 248.1±63 (1×10^3^ μl), p = 0.014, and 3.4±0.7 gm/dl vs. 3.9±0.3 m/dl, p = 0.05, respectively (c.f., Table 1).

### Comparison of hepatic expression of GHR, STAT5, IGF-1, Snail-1 and TGFBR2 proteins in the study groups

In cirrhotic patients with HCC, hepatic expression of GHR was significantly lower than that in cirrhotic patients without HCC (0.5(0–3.8) vs. 4(0.8–6), p = 0.02), and control group (0.5(0–3.8) vs. 6(4–7), p<0.001). Similar trends were observed in STAT5 and IGF-1 hepatic expression. Both were significantly lower in cirrhotic patients with HCC in comparison to those without (0.9(0–2) vs. 1.5(0–4), p = 0.02) for STAT5, and (1.5(0–6) vs. 4(3–6), p = 0.048 for IGF-1, and healthy controls (0.9(0–2) vs. 4(2–5.5), p<0.001), and 1.5(0–6) vs. 6(4.5–9), p<0.001), respectively (c.f., Table 2).

**Table 2:** Comparison of hepatic expression of GHR, STAT5, IGF-1, Snail-1 and TGFBR2 proteins in the study groups.

Variable	HCC patients G1(n = 40)	Cirrhotic patients G2(n = 40)	Healthy controls G3(n = 20)	p-value			
				Among 3 groups	p1	P2	P3
Hepatic expression:							
GHR [median (IQR)]	0.5(0–3.8)	4(0.8–6)	6(4–7)	<0.001	**0.02**	**<0.001**	**0.01**
STAT5 [(median (IQR)]	0.9(0–2)	1.5(0–4)	4(2–5.5)	0.003	**0.02**	**<0.001**	**0.03**
IGF-1[(median (IQR)]	1.5(0–6)	4(3–6)	6(4.5–9)	0.001	**0.048**	**<0.001**	**0.025**
Snail-1 [(median (IQR)]	4(3–6)	2(1–3)	1(1–2)	<0.001	**<0.001**	**<0.001**	0.228
TGFBR2 [(median (IQR)]	2.5(2–4)	4(3–6)	6(3–9)	0.028	**0.046**	**0.019**	0.213

HCC: hepatocellular carcinoma; G: group; n: number; GHR: growth hormone receptor; STAT5: signal transducer and activator of transcription 5; IGF-1: insulin like growth factor-1, TGFBR2: type2 transforming growth factor-beta receptor. Data are expressed as median (interquartile range) and compared using Kruskal Wallis test among the three groups followed by Mann Whitney test between each two groups; p1: G1 vs. G2; p2: G1 vs. G3; p3: G2 vs. G3.

Bold values denote significant results

The hepatic expression of Snail-1 was found to be significantly higher in cirrhotic patients with HCC compared to those without HCC (4(3–6) vs. 2(1–3), p<0.001) and control group (4(3–6) vs. 1(1–2), p<0.001), whereas cirrhotic patients with HCC had significantly lower hepatic expression of TGFBR2 than did cirrhotic patients without HCC (2.5(2–4) vs. 4(3–6), p = 0.046) and healthy controls (2.5(2–4) vs. 6(3–9), p = 0.019) (c.f., Table 2).

**Fig 1:**
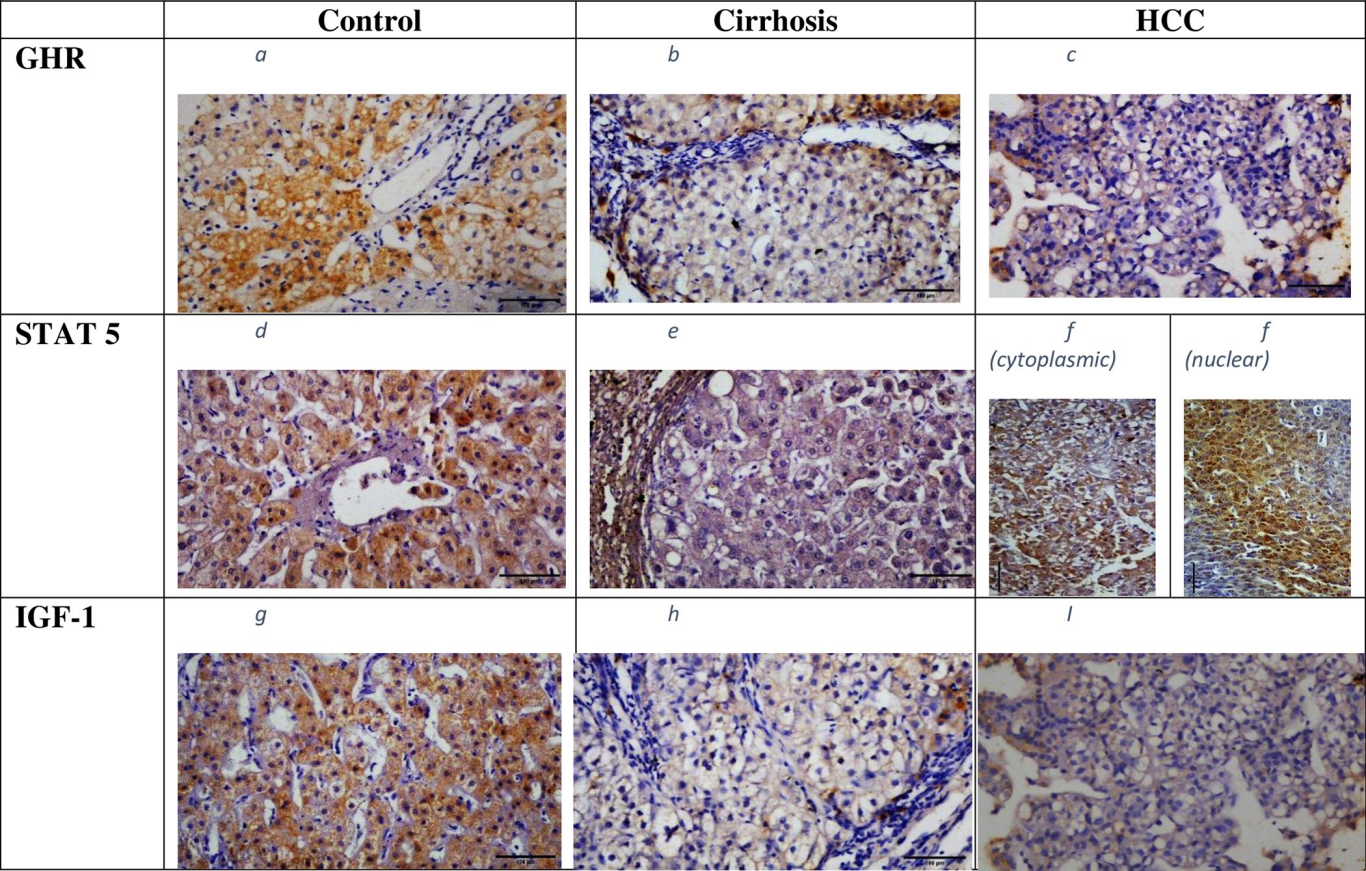
(a-i): Hepatic expression of GHR, STAT5, and IGF-1 proteins in the study groups. GHR expression: a)in healthy control: b) in cirrhotic patients; c) in HCC patients; (predominantly cytoplasmic); STAT5 expression: d) in healthy control; e) in cirrhotic patients; f) in HCC patients (cytoplasmic/nuclear) IGF-1 expression: g)in healthy controls; h) in cirrhotic patients; i) in HCC patients (predominantly cytoplasmic). Magnification 200 X scale bar 100 μm. HCC: hepatocellular carcinoma; GHR: growth hormone receptor; STAT5: signal transducer and activator of transcription5; IGF-1: insulin like growth factor-1.

Regarding the subcellular localization of GHR and IGF-1 expression, it was predominantly cytoplasmic in HCC patients. Whereas, STAT5 was found in the cytoplasm in 22(55%) cases, localized to the nucleus in 13(32.5%) cases and to both sites in 5(12.5%) cases (c.f., Fig 1A–1I).

**Fig 2:**
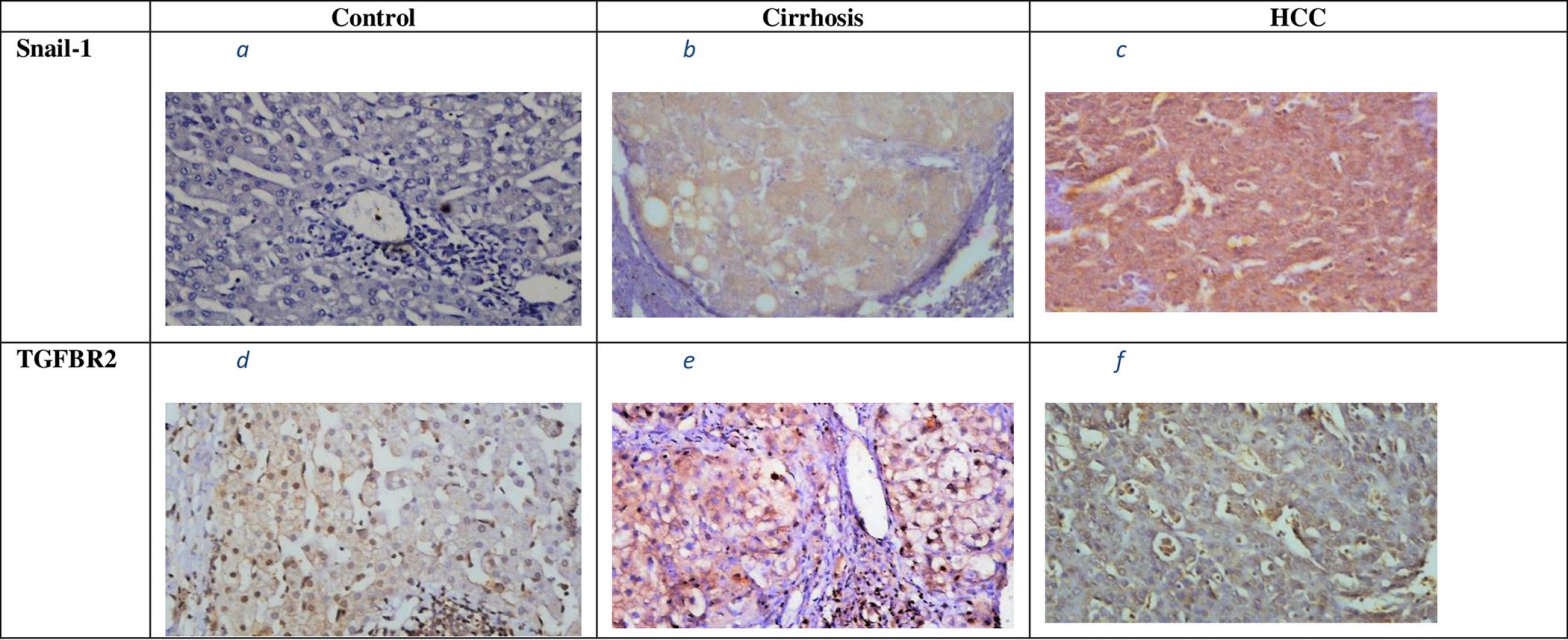
(a-f): Hepatic expression of Snail-1 and TGFBR2 proteins in the study groups. Snail-1expression: a) in healthy control: b) in cirrhotic patients; c) in HCC patients; (predominantly cytoplasmic); TGFBR2 expression: d) in healthy control; e) in cirrhotic patients; f) in HCC patients (predominantly nuclear). Magnification 200 X scale bar 100 μm. HCC: hepatocellular carcinoma; TGFBR2: type 2 transforming growth factor-beta receptor.

Snail-1 hepatic expression was mainly cytoplasmic in HCC patients, although it was mostly nuclear in case of TGFBR2 expression (c.f., Fig 2A–2F).

**Fig 3:**
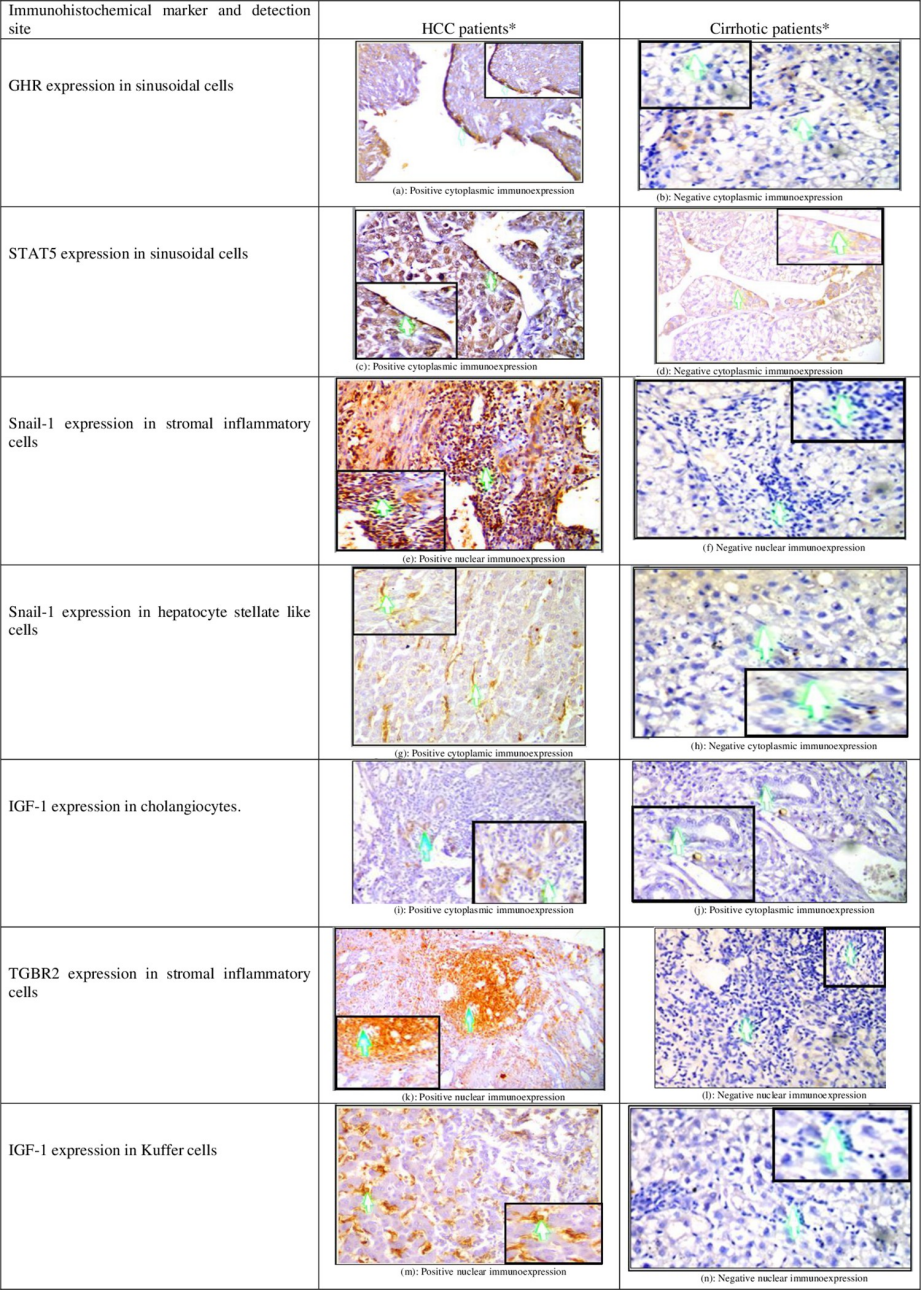
(a-n): Examples of expression of GHR, STAT5, IGF-1, Snail-1 and TGFBR2 in a variety of stromal cells of TME in HCC and cirrhotic patients. GHR: growth hormone receptor, STAT5: signal transducer and activator of transcription5; IGF-1: insulin like growth factor-1; TGFBR2: type 2 transforming growth factor-beta receptor; TME: tumor microenvironment; HCC: hepatocellular carcinoma. *: Images are presented at 20X magnification power with 200X zoom in boxes.

Moreover, we found that the expression of all these proteins in stromal cells of HCC microenvironment; including: kupffer cells (KCs), hepatic stellate cells (HSCs), liver sinusoidal endothelial cells (LSECs), Cholangiocytes, and stromal inflammatory cells was higher in HCC patients when compared to cirrhotic patients. The differences yielded statistical significance when LSECs were examined for expression of GHR (70% vs. 25%, p<0.01), STAT5 (80% vs. 15%, p<0.001) and TGFBR2 (95% vs. 10%, p>0.001). In the case of HSCs, expression of Snail-1 and TGFBR2 was significantly higher in HCC patients than cirrhotic patients (60% vs. 20%, p = 0.022, and 75% vs. 15%, p< 0.001, respectively). Similar trend was observed in stromal inflammatory cells (90% vs. 25%, p< 0.001for Snail-1 and 90% vs. 4%, p = 0.002 for TGFBR2) (data not shown) (c.f., Fig 3A–3N).

### Correlations of hepatic expression of GHR/STAT5/IGF-1 signaling pathway with different clinico-biochemical parameters, Snail-1, and TGFBR2 in cirrhotic patients

In cirrhotic patients, negative correlations were found between hepatic expression of GHR on the one hand and patient age (r = - 0.30; p = 0.03) and AFP (r = 0.31; p = 0.029) on the other one. Also a significant negative correlation was detected between hepatic expression of STAT5 and blood levels of AFP (r = - 0.29; p = 0.04). Regarding the hepatic expression of IGF-1, it was correlated positively with patient BMI (r = 0.33; p = 0.011) and negatively with patient age (r = - 0.31; p = 0.035), serum levels of liver enzymes (r = - 0.28; p = 0.03 for ALT, and r = - 0.34; p = 0.008 for AST), PT (r = - 0.27; p = 0.036), as well as serum creatinine (r = - 0.33; p = 0.013). It was notable that the hepatic expression of the three studied proteins showed significant positive correlations between them. As regards the inducers of EMT, hepatic expression of Snail-1was correlated negatively with expression of GHR, STAT5, and IGF-1 proteins (r = -0.55, p = 0.02; r = -0.47, p = 0.035, and r = -0.51, p = 0.009, respectively). On the contrary, there were positive correlations between hepatic expression of TGFBR2 and expression of all these proteins (r = 0.47, p = 0.034; r = 0.49, p = 0.023, and r = 0.57, p<0.001, respectively) (c.f., Table 3).

**Table 3:** Correlations of hepatic expression of GHR/STAT5/IGF-1 signaling pathway with different clinico-biochemical parameters, Snail-1 and TGFBR2 in cirrhotic patients.

Variable	GHR protein expression		STAT5 protein expression		IGF-1 protein expression	
	R	p-value	r	p-value	r	p-value
STAT5 hepatic expression	0.35	**0.001** †	-------	--------	-------	-------
IGF-1 hepatic expression	0.45	**0.02** †	0.53	**0.0001†**	-----	-----
Age (years)	- 0.30	**0.03** †	-0.15	0.24†	-0.31	**0.035** †
BMI (kg/m^2^)	0.20	0.12†	0.11	0.41†	0.33	**0.011** †
Smoking (Yes/No)	-0.09	0.47††	-0.05	0.72††	-0.08	0.53††
Platelets (1×10^3^/μl)	0.01	0.96†	-0.10	0.44†	0.09	0.50†
Total bilirubin (mg/dL)	-0.07	0.61†	-0.09	0.47†	0.07	0.59†
ALT (IU/L)	-0.05	0.78†	-0.05	0.72†	-0.28	**0.03** †
AST (IU/L)	-0.22	0.36†	-0.09	0.49†	-0.34	**0.008** †
Serum albumin (gm/dl)	0.17	0.20†	0.14	0.29†	0.14	0.30†
Prothrombin time (seconds)	-0.22	0.09†	-0.07	0.59†	-0.27	**0.036** †
INR	-0.12	0.38†	-0.14	0.28†	-0.18	0.231†
Serum creatinine (mg/dl)	-0.06	0.63†	-0.02	0.89†	-0.38	**0.013** †
Alpha-fetoprotein (ng/ml)	-0.31	**0.029** †	-0.29	**0.04** †	-0.23	0.08†
Child-Pugh score	-0.06	0.70†	-0.21	0.06†	-0.02	0.92†
MELD score	-0.05	0.75†	-0.15	0.36†	-0.03	0.88†
Snail-1	-0.55	**0.02†**	-0.47	**0.035** †	-0.51	**0.009** †
TGFBR2 protein	0.47	**0.034** †	0.49	**0.023** †	0.57	**<0.001** †

GHR: growth hormone receptor; STAT5: signal transducer and activator of transcription 5; IGF-1: insulin like growth factor-1; r: correlation coefficient; BMI: body mass index; kg/m^2^: kilogram/meter^2^; ALT: alanine aminotransferase; AST: aspartate transaminase; INR: international normalized ratio; MELD: model of end stage liver disease; TGFBR2: type2 transforming growth factor-beta receptor

†: Pearson’s correlation

††: Spearman’s correlation

Bold values denote significant results

### Relationship between hepatic expression of GHR/STAT5/IGF-1 signaling pathway and clinico-pathological features of the tumor in HCC patients

HCC patients with lower GHR hepatic expression exhibited significantly higher frequency of patients with AFP> 100 ng/ml (76.5% vs. 23.5%; p = 0.048); tumor size >5cm (82% vs. 18%; p = 0.02); vascular invasion (85.5% vs. 14.5%; p = 0.002), and advanced TNM stage (80.6% vs. 19.4%; p = 0.01). Also those with lower hepatic expression of STAT5 had significantly higher proportion of patients with AFP >100 ng/ml (84.8% vs. 15.2%; p = 0.006); vascular invasion (86% vs. 14; p = 0.009, and advanced pathological stage (86.7% vs. 13.3%; p = 0.007). A significantly higher proportion of patients with portal vein infiltration was found among HCC patients with lower IGF-1 hepatic expression (93.75% vs. 6.25%; p<0.001) (c.f., Table 4).

**Table 4:** Relationship between hepatic expression of GHR/STAT5/IGF-1 signaling pathway and clinico-pathological features of the tumor in cirrhotic patients with hepatocellular carcinoma.

Variable	Hepatic expression of GHR				Hepatic expression of STAT5				Hepatic expression of IGF-1			
	n.	No/low n(%)	High n(%)	p-value	n.	No/low n(%)	High n(%)	p-value	n.	No/low n(%)	High n(%)	p-value
Age (years)												
≤ 60	20	14(70)	6(30)	1	20	8(40)	12(60)	1	20	12(60)	8(40)	1
>60	20	12(60)	8(40)		20	6(30)	14(60)		20	14(70)	6(30)	
Gender												
Male	22	14(63.6)	8(36.4)	1	36	34(94.4)	2(5.6)	1	34	22(64.7)	12(35.3)	1
Female	18	12(66.7)	6(33.3)		4	4(100)	0(0)		6	4(66.7)	2(33.3)	
Child-Pugh score												
A	28	20(71.4)	8(28.6)	0.613	28	10(35.7)	18(64.3)	1	28	14(50)	14(50)	1
B+C	12	6(50)	6(50)		12	4(33.3)	8)66.7)		12	6(50)	6(50)	
MELD score												
≤12	20	16(80)	4(20)	0.350	20	8(40)	12(60)	1	20	10(50)	10(50)	1
>12	20	10(50)	10(50)		20	6(30)	14(70)		20	10(50)	10(50)	
AFP (ng/ml)												
≤100	6	2(33.3)	4(66.7)	**0.048**	7	2(28.6)	5(71.4)	**0.006**	20	10(50)	10(50)	1
>100	34	26(76.5)	8(23.5)		33	28(84.8)	5(15.2)		20	10(50)	10(50)	
Tumor number												
Single	24	18(75)	6(25)	0.356	24	8(33.3)	16(66.7)	1	24	14(58.3)	10(41.7)	0.650
Multiple	16	8(50)	8(50)		16	6(37.5)	10(62.5)		16	6(37.5)	10(62.5)	
Tumor size (cm)												
≤5	12	5(41.7)	7(58.3)	**0.02**	20	8(40)	12(60)	1	20	10(50)	10(50)	1
>5	28	23(82)	5(18)		20	6(30)	14(70)		20	10(50)	10(50)	
Vascular invasion												
No	12	4(33.3)	8(66.7)	**0.002**	11	5(45.5)	6(54.5)	**0.009**	8	2(25)	6(75)	**<0.001**
Yes	28	24(85.5)	4(14.5)		29	25(86)	4(14)		32	30(93.75)	2(6.25)	
Lymphatic permeation												
No	36	24(66.7)	12(33.3)	1	36	12(33.3)	24(66.7)	1	36	18(50)	18(50)	1
Yes	4	2(50)	2(50)		4	2(50)	2(50)		4	2(50)	2(50)	
T.N.M stage												
I	9	3(33.3)	6(66.7)	**0.01**	10	4(40)	6(60)	**0.007**	24	14(58.3)	10(41.7)	0.650
II-IV	31	25(80.6)	6(19.4)		30	26(86.7)	4(13.3)		16	6(37.5)	10(62.5)	
Okuda stage												
1	22	14(63.6)	8(36.4)	1	22	10(45.5)	12(54.5)	0.374	22	12(54.5)	10(45.5)	1
2+3	18	12(66.7)	6(33.3)		18	4(22.2)	14(77.8)		18	8(44.4)	10(55.6)	

Number of patients = 40

n.: number; GHR: growth hormone receptor; STAT5: signal transducer and activator of transcription 5; IGF-1: insulin like growth factor-1; MELD: model of end stage liver disease; AFP: alpha-fetoprotein; TNM: Tumor-Node-Metastasis. Data are expressed as proportions and percentages, and compared using Chi-square statistic or Fisher’s exact test

Bold values denote significant results

### Factors associated with overall survival in HCC patients

Herein, univariate analysis by Cox hazard model showed that age>60 years (HR (95% CI) = 2.34 (1.15–3.86); p = 0.07), vascular invasion (HR (95%CI) = 2.19(1.19–2.82); p = 0.0001); advanced TNM stage (HR (95% CI) = 3.10 (1.61–5.12); p = 0.0001), lower hepatic expression of GHR (HR (95%CI) = 3.8(1.98–5.67); p<0.0001); lower hepatic expression of STAT5 (HR(95% CI) = 1.71(1.22–2.43); p<0.01, and lower hepatic expression of IGF-1 (HR(95%CI) = 2.3(1.27–2.99); p = 0.0061) were significantly related to worse OS in HCC patients. Age > 60 years (HR(95%CI) = 2.0(1.03–3.3); p = 0.037, vascular invasion (HR(95%CI) = 3.15(1.89–10.61); p = 0.001, advanced TNM stage (HR(95%CI) = 5.32(1.63–12.43); p = 0.0014; lower GHR expression (HR(95% CI) = 3.1 (1.43–4.76); p = 0.0004), lower hepatic expression of STAT5 (HR(95%CI) = 1.45(0.96–2.33; p = 0.046) and lower hepatic expression of IGF-1 (HR(95%CI) = 1.26(0.75–2.11); p = 0.049) were found to be independent predictors of mortality in those patients when multivariate Cox analysis was used (c.f., Table 5).

**Table 5:** Univariate and multivariate analyses of risk factors related to overall survival in cirrhotic patients with hepatocellular carcinoma.

Variable	Overall survival			
	Univariate		Multivariate	
	HR (95%CI)	p-value	HR (95%CI)	p-value
Age (years) (≤ 60vs>60)	**2.34(1.15–3.86)**	**0.007**	**2.0(1.03–3.3)**	**0.037**
Gender (Male vs. Female)	0.94(0.8–1.13)	0.541		
Child-Pugh score (A vs. B+C)	1.11(0.54–1.83)	0.675		
MELD score (>12 vs. ≤12)	1.32(0.69–2.14)	0.285		
AFP (ng/ml) (≤100vs.>100)	1.59(0.81–3.12)	0.273		
Tumor number (single vs. multiple)	0.58(0.42–1.19)	0.269		
Tumor size (cm) (≤5 vs.>5)	1.37(0.65–2.43)	0.269		
Vascular invasion (No vs. Yes)	**2.19(1.19–2.82)**	**0.0001**	**3.15(1.89–10.61)**	**0.001**
Lymphatic permeation (No vs. Yes)	1.12(0.63–1.87)	0.663		
TNM stage (I vs. II-IV)	**3.10(1.11–5.12)**	**0.0001**	**5.32(1.63–12.43)**	**0.0014**
Okuda stage (1vs. 2+3)	0.83(0.49–1.51)	0.539		
GHR expression (0–4 vs. 5–12)	**3.8(1.98–5.67)**	**<0.0001**	**3.1(1.43–4.76)**	**0.0004**
STAT5 expression (0–4 vs. 5–12)	**1.71(1.22–2.43)**	**<0.01**	**1.45(0.96–2.33)**	**0.047**
IGF-1 expression (0–4 vs. 5–12)	**2.3(1.27–2.99)**	**0.0061**	**1.26(0.75–2.11)**	**0.049**

Number of patients = 40

HR: hazard ratio; CI: confidence interval; MELD: model of end stage liver disease; AFP: alpha-fetoprotein; TNM: Tumor-Node-Metastasis; GHR: growth hormone receptor; STAT5: signal transducer and activator of transcription 5; IGF-1: insulin like growth factor-1.

Bold values denote significant results

## Discussion

In spite of the progress in the management of HCC, the clinical outcome remains inadequate due to the complexity and heterogeneity of the disease, the rarity of treatment options, being diagnosed at an advanced stage, as well as, the increased rate of recurrence and distant metastasis [52]. Therefore, the recognition of the exact mechanisms which may play a role in the tumor incidence and progression, could be helpful to represent new targeted treatment approaches of HCC.

Recently, there has been increasing evidences which explore the role of GH-STAT5-1GF-1 axis in cancer development [53]. Binding of GH to its cognate receptor, is an essential step to exert its effects. In the present study, we observed that HCC patients exhibited significantly lower hepatic expression of GHR than those of cirrhosis and control groups, mostly due to hepatocellular dysfunction as a consequence of chronic liver disease, and tumor burden [54]. Our findings were compatible with the scarcely available studies in literature [55–57], albeit, GHR hepatic expression in these studies were performed by different techniques. It has been revealed that a lower GHR levels have led to a state of GH resistance which may be related to decreased GH clearance, defective synthesis of IGF-1 as a result of hepatocellular damage, and defective binding capability of GH to GHR [58]. GH mediates its pro-oncogenic effects by creating a protumorigenic milieu via various mechanisms. Elevated GH leads to accumulation of unrepaired damaged DNA, which results in increasing chromosomal instability and oncogenic aberrations [59]. GH may increase the propensity of cancer development by suppressing many tumor suppressor genes such as p53, leading to promotion of cell proliferation, and augmentation of GH role on accumulation of damaged DNA [60]. Moreover, increased GH may trigger the process of EMT by suppressing the expression of E-cadherin, which is a cell-to-cell adhesion molecule [61]. Additionally GH assists cell motility and invasion, as well as, acquiring cancer stem cell-like criteria of HCC cells, by inhibiting another tight junction protein called Claudin-1 through activation of STAT3 in HCC [62]. The effect of GH on EMT has been reported in HCC patients [63]. A significant overexpression of Snail-1 which represents a critical transcriptional repressor of E-cadherin [34], was noticed in HCC patients versus both control groups in the current study. Loss of functionality of E-cadherin has been found in different malignancies including the liver [64]. Although the distinct role of Snail-1 in triggering hepatocarcinogenesis is not wholly clear, a lot of evidence points to its vital role during malignant transformation. The tumor suppressor gene p53 hinders tumor cell invasion through the degeneration of Snail protein in HCC [65]. Co-operatively, Notch 1 and reactive oxygen species increase the level of Snail protein in hepatoma cells via induction of phosphorinositide 3- Kinase/Akt signaling pathway [66]. Yuan et al., [67] found that long non-coding UCID interacts with Snail protein to increase its stability which promots the EMT process to hasten HCC metastasis. Contrariwise, downregulated hepatic expression of TGFBR2 was seen in our HCC patients compared with the two control groups. Reduced TGFBR2 expression might result in failed cell growth arrest regulated by TGF-β which accelerates the cell oncogenesis [40]. Down-regulation of TGFBR2 is mostly due to epigenetic silencing by promotor methylation [68], in addition to TGFBR2 gene loss or mutations which are rare. Our results were consistent with those found by other investigators [44], however, others showed unchanged TGFBR2 expression in HCC patients [69]. Interestingly, we found that GHR expression negatively correlated to Snail-1 expression, but was positively correlated to TGFBR2, with statistical significance.

In HCV-related HCC patients, HCV infection leads to a production of various inflammatory and fibrotic mediators such as proinflammatory cytokines, cell death signals, and reactive oxygen species [70], as well as, hepatic stellate cells activation [71]. All these events, creates a cirrhotic microenvironment that refers to the "field cancerization", which initiates and promotes hepatocarcinogenesis [72] and probably dysregulates GHR expression. The viral core protein down-regulates CDKN1A; one of the cell cycle inhibitors leading to an aggressive type of HCC [73]. By contrast, a previous study revealed an increased expression of GHR in HCC patients as compared to healthy controls [74]. It has been reported that binding of GH to the atypically expressed GHR activates the JAK2 pathway, induces EMT, and promotes tumorigenesis [75].

Herein, we reported a significant positive correlation between hepatic expression of both GHR and IGF-1 proteins, whereas there was significant negative correlations between hepatic expression of GHR on the one hand and patient age, serum levels of AFP, vascular invasion and the TNM stage of hepatoma on the other. Also decreased hepatic expression of GHR was associated with increased serum levels of AFP, large tumor size, vascular invasion, advanced histo-pathological stage, and worse outcome. Nearly similar relations were reported by Lin et al. [57].

Many studies have been mentioned the critical roles of STAT5 proteins in the development of various solid tumors such as prostate [76], colorectal [77], breast [78], and lung [79] cancers, as well as, hematological malignancies [80]. However, studies on the role of STAT5 in HCC proved that STAT5 poses contrasting functions in different contexts, where it can act as a tumor suppressor [81, 82], or as an oncogene in other situation [83–85]. For the first time, the current study showed lower hepatic expression of STAT5 in HCC patients than cirrhotic patients and healthy volunteers, as clinical studies in this regard remains lacking. On the contrary, other investigators [83–85] reported enhanced expression of STAT5 in HCC cells that was frequently associated with tumor aggressiveness and poor clinical outcome. The oncogenic role of STAT5 in these studies was mediated through induction of cell growth and proliferation, recruitment of cancer stem cells, promotion of drug.-resistance, as well as epithelial meseuchymal transition. Furthermore, Lee et al. [83] mentioned the role of hepatitis B virus X protein in activating STAT5b in HCC patients. Meanwhile, loss of STAT5 is associated with hepatocarcinogenesis as a result of increased oncogenic STAT3 activity and induction of liver fibrosis via increased levels of TGF-β [86]. Moreover, STAT5 deficiency leads to: increased activity of oncogenic JNK1 and c-Jun (stress-activated protein kinases) [87]; attenuated expression of tumor suppressor gene p53 [88], and decreased activity of glutathione S-transferases which stimulates oxidative stress and hepatocellular damage [89]. As regard the oncogenic effects of STAT3, it is well established that activation of STAT3 promotes cell proliferation [90], and suppresses apoptosis via up-regulation of anti-apoptotic proteins [91], and down-regulation of pro-apoptotic genes [92]. It induces angiogenesis by enhancing the expression of various pro-angiogenic molecules such as vascular endothelial growth factor in the tumor microenvironment [93]. Moreover, activated STAT3 motivates the secretion of many chemokines and cytokines such as, IL-6 and IL-16 to maintain activation of immune cells in the tumor tissue [94]. STAT3 activation also regulates the expression of different cancer cells; namely CD 44 [95], and CD 133 [96] positive cells which maintain the stem cell-like characteristics in HCC cells by inducing the Notch signaling pathway [97]. Furthermore STAT3 is responsible for providing the tumor cells with the energetic demands [98]. Also it assists the motility and invasion of HCC cells by adjusting the expression of matrix metalloproteinases that cleave the extracellular matrix in the tumor microenvironment [99].

Although the activated STAT proteins localize in the nucleus [15], in the current study, STAT5 was predominantly cytoplasmic. It has been reported that STAT5 is found in both the nucleus and the cytoplasm of the cells depending upon dynamic trafficking. Its nuclear entry is induced by the importin-α3/β1 system, that is completely independent of its activation status. It continuously shuttles in and out of the nucleous through chromosome region maintenance 1-dependent and- independent pathways [100], thus unphosphorylated STAT5 could be elicited in the nucleus. Moreover, activated STAT5 may be exclusively located in the cytoplasm by inducing Scr family Kinases that leads to cytoplasmic retention of activated STAT 5 via interaction of the SH2 domain [101]. So, the oncogenic STAT5 activity may include cytoplasmic function in addition to the nuclear function through crosstalk with various signaling pathways.

The hepatic expression of STAT5 in our HCC patients was found to be inversely correlated with the tumor size, vascular invasion, TNM tumor stage, and serum level of AFP. Furthermore, lower expression of STAT5 was significantly associated with serum AFP > 100 ng/ml, vascular invasion, advanced tumor stage, and poor patient prognosis. Regarding the relation between low hepatic expression of STAT5 and vascular invasion in our HCC patients, development of HCC coexists with persistent inflammatory cells; including tumor-associated macrophages (TAM) [102]. TAMs promote EMT by producing factors such as IL-6, IL-8, TGF-β, as well as matrix metalloproteinase 2 and 9 which break down extracellular matrix to assist local invasion and metastasis of tumor cells [103]. IL-6 binds to its receptor and interacts with JAK2 leading to STAT3 activation which triggers EMT that is transcriptionally induced by Twist [104]. IL-8 is another cytokine that is secreted by TAMs in HCC; it has a role in tumor growth, survival, angiogenesis, as well as EMT via the JAK2/STAT3/Snail signaling pathway [105] and activation of CXC chemokine receptor, and CC chemokine ligand 2 [106]. Overexpression of TGF-β that secreted by TAMs interacted with different transcription factors such as Snail and Slug to induce EMT in tumor cells through down-regulation of E-cadherin expression, and up-regulation of vimentin expression [107]. Although it has been proved by RNA-sequencing analysis that STAT5 genes related to the anti-tumor immune response in TAM, Jesser et al. [108] found that loss of STAT5 in macrophages increased its ability to express tumor-promoting factors which enhanced the tumor cell migration and metastasis in vitro and in vivo. Importantly, our results showed that STAT5 expression in HCC patients was negatively correlated with Snail-1 expression, but directed to a positive correlation with TGFBR2 expression.

Another interesting finding of this study; we revealed that tissue expression of IGF-1 protein was significantly lower in HCC patients than cirrhotic patients and healthy controls. Our findings were consistent with those of previous studies [57, 109–111], but only as compared to healthy volunteers. Reduced hepatic expression of IGF-1 in HCC is multifactorial. Reduced levels of IGF-1 lead to development of GH resistance [56], decreased expression of GHR [112] due to hepatocellular damage by tumor cells [113], as well as, existence of feedback circuit at endocrine [114] and paracrine [115] loops. In this study, the tumor in HCC patients was arising on a background of HCV-associated liver cirrhosis. A lot of studies have demonstrated that liver cirrhosis by itself is responsible for decreased serum concentrations [116–118] and tissue expression [112, 119, 120] of IGF-1 protein, that have been more pronounced in patients with HCV infection than those without [20]. IGF-1 deficiency in cirrhotic patients may be related to hepatocellular dysfunction, malnutrition, oxidative damage, altered lipid metabolism, and insulin resistance [121, 122]. HCV infection triggers the expression of glucose 6-phosphatase and phosphoenol-pyruvate carboxykinase 2 leading to enhanced glucose production. Additionally, HCV infection negatively regulates the expression of glucose transporter-type 4 that is responsible for glucose uptake; therapy producing a state of insulin resistance [123], which leads to acceleration of liver fibrosis [124], and hepatocellular dysfunction that is usually followed by IGF-1 deficiency. IGF-1 deficiency has various detrimental effects. In physiological situation, liver is not a target organ for IGF-1 due to absence of its receptor in hepatocytes [125]. IGF-1 deficiency results in hepatic expression of genes encoding IGF-1 receptor and different proteins that are implicated in the inflammatory process, and acute-phase responses, and consequently oxidative damage of liver begins [126], IGF-1 may play a role in HCV-associated hepatocarcinogenesis by supporting HCV infection. IGF-1 has an inhibitory effect on lipolytic enzyme lipoprotein lipase which prevents the virus cell entry in hepatoma cells [127]. Besides, there is some sort of interaction between IGF-1 and the three oncogenic HCV proteins, including, capsid protein (protein C), and two non-structural proteins NS3 and NS5A [128].

In this study, we found a significant negative correlation between hepatic expression of IGF-1 and patient age in HCC group. During childhood, there is a greater synthesis of GH-IGF-1 axis, as a result of increased production of sex steroid hormones [129]. With age the activity of this axis shows a gradual decline to protect the organism from the harmful effects of GH on age-related attenuation of DNA repair [130]. Perhaps this could be a sensible explanation of our findings. We also observed that the hepatic expression of IGF-1 was directly related to BMI of those patients. Malnutrition is a frequent consequence of chronic liver diseases which stems from inadequate dietary intake, disturbed metabolism and malabsorption [131, 132]. It has been described that malnutrition could change the GH-IGF-1 pathway by producing GH resistance, inhibiting hepatic expression of GHR, and IGF-1 mRNA, as well as precipitating the disintegration, and reducing the bioactivity of IGF-1 [133]. However, the precise mechanism(s) remains elusive [134]. The decline of hepatic expression of IGF-1, may justify the inverse correlation between hepatic expression of IGF-1 on the one hand and some biochemical markers directly related to the severity of hepatic dysfunction; such as ALT, AST and PT on the other hand in the current study. This has promoted investigators to use the serum level of IGF-1 as an alternative to the subjective variables (ascites, hepatic encephalopathy) in Child-Pugh scoring system, for evaluating the hepatic functional reserve in HCC staging scores [135]. Also, The negative correlation between hepatic expression of IGF-1 and serum creatinine, signifies the extent of dysfunction of liver and not the kidneys, considering that chronic renal diseases usually leads to increased levels of IGF-1 [136]. Concerning the relation between hepatic expression of IGF-1 protein and the clinico-pathological characteristics of tumor, herein we observed an inverse correlation between hepatic expression of IGF-1 and vascular invasion in HCC patients. Furthermore, there was a significant association between decreased hepatic expression of IGF-1 and increased incidence of PVT in those patients. Similar finding was reported by Ikeda et al. [111]. During HCC development, increased hepatic expression of IGF-2 has been observed leaving these tissue more vulnerable to the mitogenic effects of IGF-1 [137]. IGF-1 could activate STAT5 signaling, which leads to promotion of EMT of HCC cells via downregulation of E-cadherin and upregulation of N-cadherin and vimentin [85]. Herein, the expression level of IGF-1was negatively related to Snail-1 expression, however, it was positively correlated to TGFBR2 expression.

Another valuable finding of this study concerns the increased hepatic expression of the studied proteins in a variety of cells which belong to the HCC microenvironment such as KCs, HSCs, LSECs, cholangiocytes, and stromal inflammatory cells in HCC patients. KCs are liver-resident phagocytes which play a pivotal role in different signaling pathways mediating inflammation and tumor progression [138]. Activated HSCs secret various cytokines in addition to hepatocyte growth factor that results in attenuated antitumor immunity and triggers hepatocarcinogenesis [139, 140]. LSECs contribute to occurrence of chronic liver injury and thus tumorigenesis by allowing persistence of chronic viral infections, exacerbation of fibrosis, acquisition of angiogenesis and EMT [141]. Cholangiocytes lead to liver fibrosis and hepatocarcinogesis through triggering EMT [142], and an inflammatory cytokine, II17a/f1 that activates its receptor and thus ERK dependent pathway [143]. The main stromal inflammatory cells in the HCC microenvironment are HSCs, fibroblasts, endothelial cells, adipocytes, and immune cells; which include CD8^+^ T cells, regulatory T cells, dendritic cells, and macrophages. Interactions between these cells and HCC initiate a media suitable for tumor progression [140]. Our results showed that crosstalk between GHR/STAT5/TGF1 signaling pathway and EMT inducers is required for HCC development.

Undoubtedly the current study has some limitations. First was the relatively small number of sample size. Second, the study design was retrospective. Third, STAT5 tyrosine phosphorylation status, and the functional role of each STAT5 isoform were not evaluated due to limited resources. Finally we could not rule out the role of HCV infection in downregulation of hepatic expression of GHR and its downstream pathway among HCV-associated HCC.

In conclusion, by using 1HC method, we found that down-regulation of GHR and its downstream pathway was correlated to the development of HCV-related HCC, that was associated with tumor aggressiveness and worse prognosis, irrespective of the functional status of liver. Being potent inducers of EMT, Snail-1 and TGFBR2 could be critical contributors. However, the ultimate utility of these results in practice warranted further validation by other large prospective, multi-center studies.

## Abbreviations

HCC: hepatocellular carcinoma
HCV: hepatitis C virus
IGF-1: insulin like growth factor-1
GH: growth hormone
GHR: growth hormone receptor
IGF-1R: type1 IGF receptor
IGF-2R: type 2 IGF receptor
IR: insulin receptor
IGFBP: insulin growth factor binding protein
ALS: acid-labile subunit
JAK2: Janus Kinase2
STAT: signal transducer and activator of transcription
EMT: epithelial-mesenchymal transition
TME: tumor microenvironment
TGF-β: transforming growth factor-beta
TGFBR2: type 2 TGF-β receptor
IHC: immunohistochemistry
AFP: alpha fetoprotein
MELD: model for end-stage liver disease
TNM: Tumor-Node-Metastasis
SD: standard deviation
IQR: interquartile range
OS: overall survival
HR: hazard ratio
CI: confidence interval
KCs: kupffer cells
HSCs: hepatic stellate cells
LSECs: liver sinusoidal endothelial cells
CD: cluster differentiation
TAM: tumor-associated macrophage

## References

[1] Ferlay J, Colombet M, Soerjomataram I, Mathers C, Parkin DM, Piñeros M, et al. Estimating the global cancer incidence and mortality in 2018: GLOBOCAN sources and methods. Int J Cancer. 2019; 144 (15):1941–1953. doi: 10.1002/ijc.31937 30350310

[2] Tarao K, Nozaki A, Ikeda T, Sato A, Komatsu H, Komatsu T, et al. Real impact of liver cirrhosis on the development of hepatocellular carcinoma in various liver diseases-meta-analytic assessment. Cancer Med. 2019;8(3):1054–1065. doi: 10.1002/cam4.1998 30791221 PMC6434205

[3] Abd-Elsalam S, Elwan N, Soliman H, Ziada D, El-Khalawany W, Salama M, et al. Epidemiology of liver cancer in Nile delta over a decade: A single-center study. South Asian J Cancer 2018;7(1):24–26. doi: 10.4103/sajc.sajc_82_17 29600229 PMC5865090

[4] Kouyoumjian SP, Chemaitelly H, Abu-Raddad LJ. Characterizing hepatitis C virus epidemiology in Egypt: systematic reviews, meta-analyses, and meta-regressions. Sci Rep. 2018;8(1):1661. doi: 10.1038/s41598-017-17936-4 29374178 PMC5785953

[5] Gosalia AJ, Martin P, Jones PD. Advances and future directions in the treatment of hepatocellular carcinoma. Gastroenterol Hepatol. 2017;13(7):398–410. 28867968 PMC5572970

[6] Xia F, Wu LL, Lau WY, Huan HB, Wen XD, Ma KS, et al. Adjuvant sorafenib after heptectomy for Barcelona Clinic Liver Cancer-stage C hepatocellular carcinoma patients. World J Gastroenterol. 2016;22(23):5384–5392. doi: 10.3748/wjg.v22.i23.5384 27340354 PMC4910659

[7] Bruix J, Reig M, Sherman M. Evidence-based diagnosis, staging, and treatment of patients with hepatocellular carcinoma. Gastroenterology 2016;150(4):835–853. doi: 10.1053/j.gastro.2015.12.041 26795574

[8] Quaglia A. Hepatocellular carcinoma: a review of diagnostic challenges for the pathologist. J Hepatocell Carcinoma 2018; 5:99–108. doi: 10.2147/JHC.S159808 30519546 PMC6235001

[9] Mínguez B, Tovar V, Chiang D, Villanueva A, Llovet JM. Pathogenesis of hepatocellular carcinoma and molecular therapies. Curr Opin Gastroenterol. 2009;25(3):186–194. doi: 10.1097/MOG.0b013e32832962a1 19387255

[10] Breuhahn K, Longerich T, Schirmacher P. Dysregulation of growth factor signaling in human hepatocellular carcinoma. Oncogene 2006; 25(27):3787–3800. doi: 10.1038/sj.onc.1209556 16799620

[11] Raben MS. Growth hormone. 1. Physiologic aspects. N Engl J Med. 1962; 266:31–35. doi: 10.1056/NEJM196201042660109 14038540

[12] Renaville R, Hammadi M, Portetelle D. Role of the somatotropic axis in the mammalian metabolism. Domest Anim Endocrinol. 2002;23(1–2):351–360. doi: 10.1016/s0739-7240(02)00170-4 12142251

[13] Le Roith D, Bondy C, Yakar S, Liu JL, Butler A. The somatomedin hypothesis: 2001. Endocr Rev. 2001;22(1):53–74. doi: 10.1210/edrv.22.1.0419 11159816

[14] Allard JB, Duan C. IGF-binding proteins: Why do they exist and why are there so many? Front Endocrinol. 2018;9: 117. doi: 10.3389/fendo.2018.00117 29686648 PMC5900387

[15] Mueller KM, Themanns M, Friedbichler K, Kornfeld JW, Esterbauer H, Tuckermann JP, et al. Hepatic growth hormone and glucocorticoid receptor signaling in body growth, steatosis, and metabolic liver cancer development. Mol Cell Endocrinol. 2012;361(1–2):1–11. doi: 10.1016/j.mce.2012.03.026 22564914 PMC3419266

[16] Baserga R, Peruzzi F, Reiss K. The IGF-1 receptor in cancer biology. Int J Cancer. 2003;107(6):873–877. doi: 10.1002/ijc.11487 14601044

[17] Chan JM, Stampfer MJ, Giovannucci E, Gann PH, Ma J, Wilkinson P, et al. Plasma insulin-like growth factor-I and prostate cancer risk: a prospective study. Science 1998; 279(5350):563–566. doi: 10.1126/science.279.5350.563 9438850

[18] Schernhammer ES, Holly JM, Pollak MN, Hankinson SE. Circulating levels of insulin-like growth factors, their binding proteins, and breast cancer risk. Cancer Epidemiol Biomarkers Prev. 2005;14(3):699–704. doi: 10.1158/1055-9965.EPI-04-0561 15767352

[19] Soubry A, Il’yasova D, Sedjo R, Wang F, Byers T, Rosen C, et al. Increase in circulating levels of IGF-1 and IGF-1/IGFBP-3 molar ratio over a decade is associated with colorectal adenomatous polyps. Int J Cancer. 2012;131(2):512–517. doi: 10.1002/ijc.26393 21898383 PMC3314119

[20] Su WW, Lee KT, Yeh YT, Soon MS, Wang CL, Yu ML, et al. Association of circulating insulin-like growth factor-1 with hepatocellular carcinoma: One cross-sectional correlation study. J Clin Lab Anal. 2010;24(3):195–200. doi: 10.1002/jcla.20320 20486202 PMC6647566

[21] Espelund U, Grønbæk H, Villadsen GE, Simonsen K, Vestergaard PF, Jørgensen JO, et al. The circulating IGF system in hepatocellular carcinoma: The impact of liver status and treatment. Growth Horm IGF Res. 2015;25(4):174–181. doi: 10.1016/j.ghir.2015.05.002 26068014

[22] Wang J, Li YC, Deng M, Jiang HY, Guo LH, Zhou WJ, et al. Serum insulin-like growth factor-1 and its binding protein 3 as prognostic factors for the incidence, progression, and outcome of hepatocellular carcinoma: A systematic review and meta-analysis. Oncotarget 2017; 8(46):81098–81108. doi: 10.18632/oncotarget.19186 29113370 PMC5655265

[23] Jenkins PJ, Besser M. Clinical perspective: acromegaly and cancer: a problem. J Clin Endocrinol Metab. 2001;86(7):2935–2941. doi: 10.1210/jcem.86.7.7634 11443146

[24] Swerdlow AJ, Higgins CD, Adlard P, Preece MA. Risk of cancer in patients treated with human pituitary growth hormone in the UK, 1959–89: a cohort study. Lancet 2002; 360(9329):273–277. 12147369 10.1016/s0140-6736(02)09519-3

[25] Jenkins PJ, Mukherjee A, Shalet SM. Does growth hormone cause cancer? Clin Endocrinol. 2006;64(2):115–121. doi: 10.1111/j.1365-2265.2005.02404.x 16430706

[26] Peck AR, Witkiewicz AK, Liu C, Stringer GA, Klimowicz AC, Pequignot E, et al. Loss of nuclear localized and tyrosine phosphorylated STAT5 in breast cancer predicts poor clinical outcome and increased risk of antiestrogen therapy failure. J Clin Oncol. 2011; 29(18):2448–2458. doi: 10.1200/JCO.2010.30.3552 21576635 PMC3675698

[27] Mirtti T, Leiby BE, Abdulghani J, Aaltonen E, Pavela M, Mamtani A, et al. Nuclear STAT5a/b predicts early recurrence and prostate cancer-specific death in patients treated by radical prostatectomy. Hum Pathol. 2013;44(3):310–319. doi: 10.1016/j.humpath.2012.06.001 23026195 PMC3548055

[28] Klupp F, Diers J, Kahlert C, Neumann L, Halama N, Franz C, et al. Expressional STAT3/STAT5 ratio is an independent prognostic marker in colon carcinoma. Ann Surg Oncol. 2015;22 (Suppl 3):S1548–S1555. doi: 10.1245/s10434-015-4485-4 25773877

[29] Ferbeyre G, Moriggl R. The role of STAT5 transcription factors as tumor suppressors or oncogenes. Biochim Biophys Acta. 2011;1815(1):104–114. doi: 10.1016/j.bbcan.2010.10.004 20969928

[30] Thiery JP. Epithelial-mesenchymal transition in tumour progression. Nat Rev Cancer. 2002;2:442–454. 12189386 10.1038/nrc822

[31] Peinado H, Lavotshkin S, Lyden D. The secreted factors responsible for pre-metastatic niche formation: old sayings and new thoughts. Semin Cancer Biol. 2011;21:139–146. doi: 10.1016/j.semcancer.2011.01.002 21251983

[32] Beavon IR. The E-cadherin-catenin complex in tumour metastasis: structure, function and regulation. Eur J Cancer. 2000;36:1607–1620. doi: 10.1016/s0959-8049(00)00158-1 10959047

[33] Tsai JH, Donaher JL, Murphy DA, Chau S, Yang J. Spatiotemporal regulation of epithelial-mesenchymal transition is essential for squamous cell carcinoma metastasis. Cancer Cell 2012;22(6):725–736. doi: 10.1016/j.ccr.2012.09.022 23201165 PMC3522773

[34] Osley MA, Fleming AB, Kao CF. Histone ubiquitylation and the regulation of transcription. Results Probl Cell Differ. 2006;41:47–75. doi: 10.1007/400_006 16909890

[35] Roy HK, Iversen P, Hart J, Liu Y, Koetsier JL, Kim Y, et al. Down-regulation of SNAIL suppresses MIN mouse tumorigenesis: modulation of apoptosis, proliferation, and fractal dimension. Mol Cancer Ther. 2004;3:1159–1165. 15367710

[36] Lyons JG, Patel V, Roue NC, Fok SY, Soon LL, Halliday GM, et al. Snail up-regulates proinflammatory mediators and inhibits differentiation in oral keratinocytes. Cancer Res. 2008;68:4525–4530. doi: 10.1158/1078-0432.CCR-07-6735 18559496 PMC2631428

[37] Wang H, Zhang G, Zhang H, Zhang F, Zhou B, Ning F, et al. Acquisition of epithelial-mesenchymal transition phenotype and cancer stem cell-like properties in cisplatin-resistant lung cancer cells through AKT/β-catenin/Snail signaling pathway. Eur J Pharmacol. 2014;723:156–166. doi: 10.1016/j.ejphar.2013.12.004 24333218

[38] Jing Y, Han Z, Zhang S, Liu Y, Wei L. Epithelial-mesenchymal transition in tumor microenvironment. Cell Biosci. 2011; 31;1:29. doi: 10.1186/2045-3701-1-29 21880137 PMC3179439

[39] Derynck R, Zhang YE. Smad-dependent and Smad-independent pathways in TGF-beta family signalling. Nature 2003;425:577–584. doi: 10.1038/nature02006 14534577

[40] Ikushima H, Miyazono K. TGF-beta signalling: a complex web in cancer progression. Nat Rev Cancer 2010;10:415–424. doi: 10.1038/nrc2853 20495575

[41] Xu JB, Bao Y, Liu X, Liu Y, Huang S, Wang JC. Defective expression of transforming growth factor -beta type II receptor (TGFBR2) in the large cell variant of non-small cell lung carcinoma. Lung Cancer 2007;58(1):36–43. doi: 10.1016/j.lungcan.2007.04.019 17566598

[42] Yang H, Zhang H, Zhong Y, Wang Q, Yang L, Kang H, et al. Concomitant underexpression of TGFBR2 and overexpression of hTERT are associated with poor prognosis in cervical cancer. Sci Rep. 2017;7:41670–41689. doi: 10.1038/srep41670 28195144 PMC5307321

[43] Zhou H, Wu G, Ma X, Xiao J, Yu G, Yang C, et al. Attenuation of TGFBR2 expression and tumour progression in prostate cancer involve diverse hypoxia-regulated pathways. J Exp Clin Cancer Res. 2018;37:89–101. doi: 10.1186/s13046-018-0764-9 29699590 PMC5921809

[44] Mamiya T, Yamazaki K, Masugi Y, Mori T, Effendi K, Du W, et al. Reduced transforming growth factor-beta receptor II expression in hepatocellular carcinoma correlates with intrahepatic metastasis. Lab Invest. 2010;90(9):1339–1345. doi: 10.1038/labinvest.2010.105 20531292

[45] Bruix J, Sherman M; American Association for the Study of Liver Diseases. Management of hepatocellular carcinoma: an update. Hepatology 2011;53(3):1020–1022. doi: 10.1002/hep.24199 21374666 PMC3084991

[46] Sherman M. Histological diagnosis of early hepatocellular carcinoma. Hepatology 2011;53(5):1427–1429. doi: 10.1002/hep.24332 21452287

[47] Bedossa P, Poynard T. An algorithm for the grading of activity in chronic hepatitis C. The METAVIR Cooperative Study Group. Hepatology 1996;24(2):289–293. doi: 10.1002/hep.510240201 8690394

[48] Pugh RN, Murray-Lyon IM, Dawson JL, Pietroni MC, Williams R. Transection of the oesophagus for bleeding oesophageal varices. Br J Surg. 1973; 60(8):646–649. doi: 10.1002/bjs.1800600817 4541913

[49] Kamath PS, Wiesner RH, Malinchoc M, Kremers W, Therneau TM, Kosberg CL, et al. A model to predict survival in patients with end-stage liver disease. Hepatology 2001; 33(2):464–470. doi: 10.1053/jhep.2001.22172 11172350

[50] Kee KM, Wang JH, Lin CY, Wang CC, Cheng YF, Lu SN. Validation of the 7th edition TNM staging system for hepatocellular carcinoma: an analysis of 8,828 patients in a single medical center. Dig Dis Sci. 2013;58(9):2721–2728. doi: 10.1007/s10620-013-2716-8 23703450

[51] Okuda K, Obata H, Nakajima Y, Ohtsuki T, Okazaki N, Ohnishi K. Prognosis of primary hepatocellular carcinoma. Hepatology 1984;4(1 Suppl):3s–6s. doi: 10.1002/hep.1840040703 6319264

[52] Sempokuya T, Wong LL. Ten-year survival and recurrence of hepatocellular cancer. Hepatoma Res. 2019;5:38. doi: 10.20517/2394-5079.2019.013 31701016 PMC6836870

[53] Werner H, Laron Z. Role of the GH-IGF1 system in progression of cancer. Mol Cell Endocrinol. 2020;518:111003. doi: 10.1016/j.mce.2020.111003 32919021

[54] Li L, Mo F, Hui EP, Chan SL, Koh J, Tang NLS, et al. The association of liver function and quality of life of patients with liver cancer. BMC Gastroenterol. 2019;19:66. doi: 10.1186/s12876-019-0984-2 31046687 PMC6498612

[55] Chang TC, Lin JJ, Yu SC, Chang TJ. Absence of growth-hormone receptor in hepatocellular carcinoma and cirrhotic liver. Hepatology 1990;11(1):123–126. doi: 10.1002/hep.1840110121 2153093

[56] Liu JP, Wang HT, Ou QJ, Lu YT, Lu HP, Gao JH, et al. [Expression of growth hormone receptor in hepatocellular carcinoma and its significance]. Ai Zheng 2003;22(3):298–301 12654191

[57] Lin CC, Liu TW, Yeh ML, Tsai YS, Tsai PC, Huang CF, et al. Significant down-regulation of growth hormone receptor expression revealed as a new unfavorable prognostic factor in hepatitis C virus-related hepatocellular carcinoma. Clin Mol Hepatol. 2021;27(2):313–328. doi: 10.3350/cmh.2020.0247 33317258 PMC8046631

[58] Baruch Y, Assy N, Amit T, Krivoy N, Strickovsky D, Orr ZS, et al. Spontaneous pulsatility and pharmacokinetics of growth hormone in liver cirrhotic patients. J Hepatol. 1998;29(4):559–564. doi: 10.1016/s0168-8278(98)80150-5 9824264

[59] Bayram F, Bitgen N, Donmez-Altuntas H, Cakir I, Hamurcu Z, Sahin F, et al. Increased genome instability and oxidative DNA damage and their association with IGF-1 levels in patients with active acromegaly. Growth Horm IGF Res. 2014;24:29–34. doi: 10.1016/j.ghir.2013.12.002 24382376

[60] Chesnokova V, Melmed S. Growth hormone in the tumor microenvironment. Arch Endocrinol Metab. 2019;63(6):568–575. doi: 10.20945/2359-3997000000186 31939481 PMC7025769

[61] Wang JJ, Chong QY, Sun XB, You ML, Pandey V, Chen YJ, et al. Autocrine hGH stimulates oncogenicity, epithelial-mesenchymal transition and cancer stem cell-like behavior in human colorectal carcinoma. Oncotarget 2017;8:103900–103918. doi: 10.18632/oncotarget.21812 29262609 PMC5732775

[62] Chen YJ, You ML, Chong QY, Pandey V, Zhuang QS, Liu DX, et al. Autocrine human growth hormone promotes invasive and cancer stem cell-like behavior of hepatocellular carcinoma cells by STAT3 dependent inhibition of CLAUDIN-1 expression. Int J Mol Sci. 2017;18:1274–1294. doi: 10.3390/ijms18061274 28617312 PMC5486096

[63] Kong X, Wu W, Yuan Y, Pandey V, Wu Z, Lu X, et al. Human growth hormone and human prolactin function as autocrine/paracrine promoters of progression of hepatocellular carcinoma. Oncotarget 2016;7:29465–29479. doi: 10.18632/oncotarget.8781 27102295 PMC5045410

[64] Berx G, van Roy F. Involvement of members of the cadherin superfamily in cancer. Cold Spring Harb Perspect Biol. 2009;1(6):a003129. doi: 10.1101/cshperspect.a003129 20457567 PMC2882122

[65] Lim SO, Kim H, Jung G. p53 inhibits tumor cell invasion via the degradation of Snail protein in hepatocellular carcinoma. FEBS Lett. 2010; 584(11):2231–2236. doi: 10.1016/j.febslet.2010.04.006 20385133

[66] Kim HS, Jung G. Notch1 increases Snail expression under high reactive oxygen species conditions in hepatocellular carcinoma cells. Free Radic Res. 2014;48(7):806–813. doi: 10.3109/10715762.2014.909595 24684482

[67] Yuan S, Si W, Zhuang K, Li Y, Zhang Y, Liu J, et al. LncRNA UCID promotes hepatocellular carcinoma metastasis via stabilization of Snail. Onco Targets Ther. 2021;14:725–736. doi: 10.2147/OTT.S277951 33536764 PMC7850577

[68] Zhao H, Shiina H, Greene KL, Li LC, Tanaka Y, Kishi H, et al. CpG methylation at promoter site -140 inactivates TGF beta2 receptor gene in prostate cancer. Cancer 2005;104:44–52. doi: 10.1002/cncr.21135 15895377

[69] Abou-Shady M, Baer HU, Friess H, Berberat P, Zimmermann A, Graber H, et al. Transforming growth factor betas and their signaling receptors in human hepatocellular carcinoma. Am J Surg. 1999;177:209–215. doi: 10.1016/s0002-9610(99)00012-4 10219856

[70] Lee YA, Wallace MC, Friedman SL. Pathobiology of liver fibrosis: a translational success story. Gut 2015;64:830–841. doi: 10.1136/gutjnl-2014-306842 25681399 PMC4477794

[71] Bataller R, Paik YH, Lindquist JN, Lemasters JJ, Brenner DA. Hepatitis C virus core and nonstructural proteins induce fibrogenic effects in hepatic stellate cells. Gastroenterology 2004;126:529–540. doi: 10.1053/j.gastro.2003.11.018 14762790

[72] Aihara T, Noguchi S, Sasaki Y, Nakano H, Imaoka S. Clonal analysis of regenerative nodules in hepatitis C virus-induced liver cirrhosis. Gastroenterology 1994;107:1805–1811. doi: 10.1016/0016-5085(94)90824-9 7958695

[73] Machida K, Liu JC, McNamara G, Levine A, Duan L, Lai MM. Hepatitis C virus causes uncoupling of mitotic checkpoint and chromosomal polyploidy through the Rb pathway. J Virol. 2009;83:12590–12600. doi: 10.1128/JVI.02643-08 19793824 PMC2786750

[74] García-Caballero T, Mertani HM, Lambert A, Gallego R, Fraga M, Pintos E, et al. Increased expression of growth hormone and prolactin receptors in hepatocellular carcinomas. Endocrine 2000;12 (3):265–271. doi: 10.1385/ENDO:12:3:265 10963047

[75] Conway-Campbell BL, Wooh JW, Brooks AJ, Gordon D, Brown RJ, Lichanska AM, et al. Nuclear targeting of the growth hormone receptor results in dysregulation of cell proliferation and tumorigenesis. Proc Natl Acad Sci U S A. 2007;104(33):13331–1336. doi: 10.1073/pnas.0600181104 17690250 PMC1948913

[76] Gu L, Liao Z, Hoang DT, Dagvadorj A, Gupta S, Blackmon S, et al. Pharmacologic inhibition of Jak2-Stat5 signaling by Jak2 inhibitor AZD1480 potently suppresses growth of both primary and castrate-resistant prostate cancer. Clin Cancer Res. 2013;19:5658–5674. doi: 10.1158/1078-0432.CCR-13-0422 23942095 PMC6021137

[77] Zhang L, Li J, Li L, Zhang J, Wang X, Yang C, et al. IL-23 selectively promotes the metastasis of colorectal carcinoma cells with impaired Socs3 expression via the STAT5 pathway. Carcinogenesis 2014;35:1330–1340. doi: 10.1093/carcin/bgu017 24464786

[78] Lee GH, Yoo KC, An Y, Lee HJ, Lee M, Uddin N, et al. FYN promotes mesenchymal phenotypes of basal type breast cancer cells through STAT5/NOTCH2 signaling node. Oncogene 2018;37:1857–1868. doi: 10.1038/s41388-017-0114-y 29348460

[79] Jung YY, Shanmugam MK, Narula AS, Kim C, Lee JH, Namjoshi OA, et al. Oxymatrine attenuates tumor growth and deactivates STAT5 signaling in a lung cancer xenograft model. Cancers (Basel) 2019;11(1):49. 10.3390/cancers11010049 PMC6356594 30621055

[80] Kollmann S, Grundschober E, Maurer B, Warsch W, Grausenburger R, Edlinger L, et al. Twins with different personalities: STAT5B-but not STAT5A-has a key role in BCR/ABL-induced leukemia. Leukemia 2019;33:1583–1597. doi: 10.1038/s41375-018-0369-5 30679796 PMC6755975

[81] Hosui A, Kimura A, Yamaji D, Zhu BM, Na R, Hennighausen L. Loss of STAT5 causes liver fibrosis and cancer development through increased TGF-{beta} and STAT3 activation. J Exp Med. 2009;206:819–831. doi: 10.1084/jem.20080003 19332876 PMC2715112

[82] Yu JH, Zhu BM, Riedlinger G, Kang K, Hennighausen L. The liver-specific tumor suppressor STAT5 controls expression of the reactive oxygen species-generating enzyme NOX4 and the proapoptotic proteins PUMA and BIM in mice. Hepatology 2012;56:2375–2386. doi: 10.1002/hep.25900 22711600 PMC3505809

[83] Lee TK, Man K, Poon RT, Lo CM, Yuen AP, Ng IO, et al. Signal transducers and activators of transcription 5b activation enhances hepatocellular carcinoma aggressiveness through induction of epithelial-mesenchymal transition. Cancer Res. 2006;66:9948–9956. doi: 10.1158/0008-5472.CAN-06-1092 17047057

[84] Fu B, Meng W, Zhao H, Zhang B, Tang H, Zou Y, et al. GRAM domain-containing protein 1A (GRAMD1A) promotes the expansion of hepatocellular carcinoma stem cell and hepatocellular carcinoma growth through STAT5. Sci Rep. 2016;6:31963. doi: 10.1038/srep31963 27585821 PMC5009375

[85] Zhao C, Wang Q, Wang B, Sun Q, He Z, Hong J, et al. IGF-1 induces the epithelial-mesenchymal transition via Stat5 in hepatocellular carcinoma. Oncotarget 2017;8:111922–111930. doi: 10.18632/oncotarget.22952 29340101 PMC5762369

[86] Bataller R, Brenner DA. Liver fibrosis. J Clin Invest. 2005;115:209–218. doi: 10.1172/JCI24282 15690074 PMC546435

[87] Zanke BW, Boudreau K, Rubie E, Winnett E, Tibbles LA, Zon L, et al. The stress-activated protein kinase pathway mediates cell death following injury induced by cis-platinum, UV irradiation or heat. Curr Biol. 1996;6:606–613. doi: 10.1016/s0960-9822(02)00547-x 8805279

[88] Friedbichler K, Themanns M, Mueller KM, Schlederer M, Kornfeld JW, Terracciano LM, et al. Growth-hormone-induced signal transducer and activator of transcription 5 signaling causes gigantism, inflammation, and premature death but protects mice from aggressive liver cancer. Hepatology 2012;55:941–952. 10.1002/hep.24765 22031092

[89] Zhou YC, Waxman DJ. STAT5b down-regulates peroxisome proliferator-activated receptor alpha transcription by inhibition of ligand-independent activation function region-1 trans-activation domain. J Biol Chem. 1999;274:29874–29882. doi: 10.1074/jbc.274.42.29874 10514468

[90] Bromberg JF, Wrzeszczynska MH, Devgan G, Zhao Y, Pestell RG, Albanese C, et al. STAT3 as an oncogene. Cell 1999;98:295–303. doi: 10.1016/s0092-8674(00)81959-5 10458605

[91] Gritsko T, Williams A, Turkson J, Kaneko S, Bowman T, Huang M, et al. Persistent activation of STAT3 signaling induces survivin gene expression and confers resistance to apoptosis in human breast cancer cells. Clin Cancer Res. 2006;12(1):11–19. doi: 10.1158/1078-0432.CCR-04-1752 16397018

[92] Timofeeva OA, Tarasova NI, Zhang X, Chasovskikh S, Cheema AK, Wang H, et al. STAT3 suppresses transcription of proapoptotic genes in cancer cells with the involvement of its N-terminal domain. Proc Natl Acad Sci U S A. 2013;110(4):1267–1272. doi: 10.1073/pnas.1211805110 23288901 PMC3557078

[93] Carbajo-Pescador S, Ordoñez R, Benet M, Jover R, García-Palomo A, Mauriz JL, et al. Inhibition of VEGF expression through blockade of Hif1alpha and STAT3 signalling mediates the anti-angiogenic effect of melatonin in HepG2 liver cancer cells. Br J Cancer. 2013;109(1):83–91. 23756865 10.1038/bjc.2013.285 PMC3708553

[94] Yu H, Pardoll D, Jove R. STATs in cancer inflammation and immunity: a leading role for STAT3. Nat Rev Cancer. 2009;9(11):798–809. doi: 10.1038/nrc2734 19851315 PMC4856025

[95] Toh TB, Lim JJ, Hooi L, Rashid MBMA, Chow. Targeting Jak/Stat pathway as a therapeutic strategy against SP/CD44+ tumorigenic cells in Akt/β-catenin-driven hepatocellular carcinoma. J Hepatol. 2020;72(1):104–118. doi: 10.1016/j.jhep.2019.08.035 31541681

[96] Won C, Kim BH, Yi EH, Choi KJ, Kim EK, Jeong JM, et al. Signal transducer and activator of transcription 3-mediated CD133 up-regulation contributes to promotion of hepatocellular carcinoma. Hepatology 2015;62(4):1160–1173. doi: 10.1002/hep.27968 26154152 PMC5049669

[97] Xiong S, Wang R, Chen Q, Luo J, Wang J, Zhao Z, et al. Cancer-associated fibroblasts promote stem cell-like properties of hepatocellular carcinoma cells through IL-6/STAT3/Notch signaling. Am J Cancer Res. 2018;8(2):302–316. 29511600 PMC5835697

[98] Huynh J, Chand A, Gough D, Ernst M. Therapeutically exploiting STAT3 activity in cancer—using tissue repair as a road map. Nat Rev Cancer. 2019;19(2):82–96. doi: 10.1038/s41568-018-0090-8 30578415

[99] Li WC, Ye SL, Sun RX, Liu YK, Tang ZY, Kim Y, et al. Inhibition of growth and metastasis of human hepatocellular carcinoma by antisense oligonucleotide targeting signal transducer and activator of transcription 3. Clin Cancer Res. 2006;12(23):7140–7148. doi: 10.1158/1078-0432.CCR-06-0484 17145839

[100] Shin HY, Reich NC. Dynamic trafficking of STAT5 depends on an unconventional nuclear localization signal. J Cell Sci 2013, 126: 3333–3343 doi: 10.1242/jcs.123042 23704351 PMC3730243

[101] Chatain N, Ziegler P, Fahrenkamp D, Jost E, Moriggl R, Schmitz-Van de Leur H, et al. Scr family kinases mediate cytoplasmic retention of activated STAT5 in BCR-ABL positive cells. Oncogene 2013, 32: 3587–3597 22926520 10.1038/onc.2012.369

[102] Kim J, Bae JS. Tumor-associated macrophages and neutrophils in tumor microenvironment. Mediators Inflamm. 2016;2016:6058147. doi: 10.1155/2016/6058147 26966341 PMC4757693

[103] Chanmee T, Ontong P, Konno K, Itano N. Tumor-associated macrophages as major players in the tumor microenvironment. Cancers (Basel) 2014; 6(3):1670–1690. doi: 10.3390/cancers6031670 25125485 PMC4190561

[104] Zhang C, Guo F, Xu G, Ma J, Shao F. STAT3 cooperates with Twist to mediate epithelial-mesenchymal transition in human hepatocellular carcinoma cells. Oncol Rep. 2015;33(4):1872–1882. doi: 10.3892/or.2015.3783 25653024

[105] Fu XT, Dai Z, Song K, Zhang ZJ, Zhou ZJ, Zhou SL, et al. Macrophage-secreted IL-8 induces epithelial-mesenchymal transition in hepatocellular carcinoma cells by activating the JAK2/STAT3/Snail pathway. Int J Oncol. 2015;46(2):587–596. doi: 10.3892/ijo.2014.2761 25405790

[106] Huang W, Chen Z, Zhang L, Tian D, Wang D, Fan D, et al. Interleukin-8 induces expression of FOXC1 to promote transactivation of CXCR1 and CCL2 in hepatocellular carcinoma cell lines and formation of metastases in mice. Gastroenterology 2015;149(4):1053–1067.e14. doi: 10.1053/j.gastro.2015.05.058 26065367

[107] Moon H, Ju HL, Chung SI, Cho KJ, Eun JW, Nam SW, et al. transforming growth factor-beta promotes liver tumorigenesis in mice via up-regulation of Snail. Gastroenterology 2017;153(5):1378–1391.e6. 28734833 10.1053/j.gastro.2017.07.014

[108] Jesser EA, Brady NJ, Huggins DN, Witschen PM, O’Connor CH, Schwertfeger KL. STAT5 is activated in macrophages by breast cancer cell-derived factors and regulates macrophage function in the tumor microenvironment. Breast Cancer Res. 2021;23(1):104. doi: 10.1186/s13058-021-01481-0 34743736 PMC8573892

[109] Huynh H, Chow PK, Ooi LL, Soo KC. A possible role for insulin-like growth factor-binding protein-3 autocrine/paracrine loops in controlling hepatocellular carcinoma cell proliferation. Cell Growth Differ. 2002;13:115–122. 11959812

[110] Adamek A, Kasprzak A, Mikoś H, Przybyszewska W, Seraszek-Jaros A, Czajka A, et al. The insulin-like growth factor-1 and expression of its binding protein-3 in chronic hepatitis C and hepatocellular carcinoma. Oncol Rep. 2013;30:1337–1345 doi: 10.3892/or.2013.2546 23784592

[111] Ikeda Y, Kajiyama K, Yamashita Y, Ikegami T, Uchiyama H, Soejima Y, et al. Differential expression of insulin-like growth factor 1 in human primary liver cancer. Fukuoka Igaku Zasshi. 2013;104:334–338. 24511663

[112] Su TS, Liu WY, Han SH, Jansen M, Yang-Fen TL, P’eng FK, et al. Transcripts of the insulin-like growth factors I and II in human hepatoma. Cancer Res. 1989;49(7):1773–1777. 2466561

[113] Donaghy AJ, Delhanty PJ, Ho KK, Williams R, Baxter RC. Regulation of the growth hormone receptor/binding protein, insulin-like growth factor ternary complex system in human cirrhosis. J Hepatol. 2002;36(6):751–758. doi: 10.1016/s0168-8278(02)00049-1 12044524

[114] Strewler GJ. Humoral manifestations of malignancy. In: Wilson JD, Foster DW, Kronenberg HH, and Larsen PR, editors. Endocrinology, 9 th Edition, WB Saunders Company, Philadelphia 1998, pp. 1693–1710.

[115] Rogler CE, Chisari FV. Cellular and molecular mechanisms of hepatocarcinogenesis. Semin Liver Dis. 1992;12:265–278. doi: 10.1055/s-2007-1007398 1439879

[116] Vyzantiadis T, Theodoridou S, Giouleme O, Harsoulis P, Evgenidis N, Vyzantiadis A. Serum concentrations of insulin-like growth factor-I (IGF-I) in patients with liver cirrhosis. Hepatogastroenterology 2003;50:814–816. 12828091

[117] Wu YL, Ye J, Zhang S, Zhong J, Xi RP. Clinical significance of serum IGF-I, IGF-II and IGFBP-3 in liver cirrhosis. World J Gastroenterol. 2004;10:2740–2743. doi: 10.3748/wjg.v10.i18.2740 15309731 PMC4572205

[118] Adamek A, Kasprzak A, Seraszek A, Mikoś H, Bura A, Mozer-Lisewska I. Alterations of insulin-like growth factor I (IGF-I) and estradiol serum levels in chronic hepatitis C. Contemp Oncol (Pozn). 2012;16:234–239. 23788886 10.5114/wo.2012.29291 PMC3687405

[119] Luo SM, Tan WM, Deng WX, Zhuang SM, Luo JW. Expression of albumin, IGF-1, IGFBP-3 in tumor tissues and adjacent non-tumor tissues of hepatocellular carcinoma patients with cirrhosis. World J Gastroenterol. 2005;11(27):4272–4276. doi: 10.3748/wjg.v11.i27.4272 16015705 PMC4615458

[120] Hribal ML, Procopio T, Petta S, Sciacqua A, Grimaudo S, Pipitone RM, et al. Insulin-like growth factor-I, inflammatory proteins, and fibrosis in subjects with nonalcoholic fatty liver disease. J Clin Endocrinol Metab. 2013;98:E304–E308. doi: 10.1210/jc.2012-3290 23316084

[121] García-Fernández M, Delgado G, Puche JE, González-Barón S, Castilla Cortázar I. Low doses of insulin-like growth factor-I improve insulin resistance, lipid metabolism, and oxidative damage in aging rats. Endocrinology 2008;149(5):2433–2442. doi: 10.1210/en.2007-1190 18187555

[122] Aguirre GA, De Ita JR, de la Garza RG, Castilla-Cortazar I. Insulin-like growth factor-1 deficiency and metabolic syndrome. J Transl Med. 2016;14:3. doi: 10.1186/s12967-015-0762-z 26733412 PMC4702316

[123] Bose SK, Shrivastava S, Meyer K, Ray RB, Ray R. Hepatitis C virus activates the mTOR/S6K1 signaling pathway in inhibiting IRS-1 function for insulin resistance. J Virol. 2012;86:6315–6322. doi: 10.1128/JVI.00050-12 22457523 PMC3372214

[124] Mohamed AA, Loutfy SA, Craik JD, Hashem AG, Siam I. Chronic hepatitis C genotype-4 infection: role of insulin resistance in hepatocellular carcinoma. Virol J. 2011;8:496. doi: 10.1186/1743-422X-8-496 22044490 PMC3218090

[125] Massagué J, Czech MP. The subunit structures of two distinct receptors for insulin-like growth factors I and II and their relationship to the insulin receptor. J Biol Chem. 1982;257:5038–5045. 6279656

[126] Lara-Diaz VJ, Castilla-Cortazar I, Martín-Estal I, García-Magariño M, Aguirre GA, Puche JE, et al. IGF-1 modulates gene expression of proteins involved in inflammation, cytoskeleton, and liver architecture. J Physiol Biochem. 2017;73:245–258. doi: 10.1007/s13105-016-0545-x 28124277 PMC5399066

[127] Maillard P, Walic M, Meuleman P, Roohvand F, Huby T, Le Goff W, et al. Lipoprotein lipase inhibits hepatitis C virus (HCV) infection by blocking virus cell entry. PLoS One 2011;6(10):e26637. doi: 10.1371/journal.pone.0026637 22039521 PMC3198807

[128] Kasprzak A, Adamek A, Przybyszewska W, Szaflarski W, Sterzyńska K, Seraszek A, et al. Expression of IGF-I and viral proteins (C, NS3, NS5A) in livers of patients with chronic HCV infection. Adv Clin Exp Med. 2011;20:263–273.

[129] Giustina A, Scalvini T, Tassi C, Desenzani P, Poiesi C, Wehrenberg WB, et al. Maturation of the regulation of growth hormone secretion in young males with hypogonadotropic hypogonadism pharmacologically exposed to progressive increments in serum testosterone. J Clin Endocrinol Metab. 1997;82(4):1210–1219. doi: 10.1210/jcem.82.4.3871 9100598

[130] Chesnokova V, Zonis S, Barrett R, Kameda H, Wawrowsky K, Ben-Shlomo A, et al. Excess growth hormone suppresses DNA damage repair in epithelial cells. JCI Insight. 2019;4(3):e125762. doi: 10.1172/jci.insight.125762 30728323 PMC6413789

[131] Cheung K, Lee SS, Raman M. Prevalence and mechanisms of malnutrition in patients with advanced liver disease, and nutrition management strategies. Clin Gastroenterol Hepatol. 2012;10:117–125. doi: 10.1016/j.cgh.2011.08.016 21893127

[132] Chapman B, Sinclair M, Gow PJ, Testro AG. Malnutrition in cirrhosis: More food for thought. World J Hepatol. 2020;12(11):883–896. doi: 10.4254/wjh.v12.i11.883 33312416 PMC7701970

[133] Fuentes EN, Einarsdottir IE, Valdes JA, Alvarez M, Molina A, Björnsson BT. Inherent growth hormone resistance in the skeletal muscle of the fine flounder is modulated by nutritional status and is characterized by high contents of truncated GHR, impairment in the JAK2/STAT5 signaling pathway, and low IGF-I expression. Endocrinology 2012;153(1):283–294. doi: 10.1210/en.2011-1313 22028448

[134] Ban B, Zhao Q. Nutritional regulation of growth hormone/insulin-like growth factor-1 axis. Nutri Food Sci Int J. 2018; 7(5): 154–156.‏

[135] Kaseb AO, Xiao L, Hassan MM, Chae YK, Lee JS, Vauthey JN, et al. Development and validation of insulin-like growth factor-1 score to assess hepatic reserve in hepatocellular carcinoma. J Natl Cancer Inst. 2014; 106(5):dju088. doi: 10.1093/jnci/dju088 24815863 PMC4085880

[136] Teppala S, Shankar A, Sabanayagam C. Association between IGF-1 and chronic kidney disease among US adults. Clin Exp Nephrol. 2010;14:440–444. doi: 10.1007/s10157-010-0307-y 20567872

[137] Qiu LW, Yao DF, Zong L, Lu YY, Huang H, Wu W, et al. Abnormal expression of insulin-like growth factor-II and its dynamic quantitative analysis at different stages of hepatocellular carcinoma development. Hepatobiliary Pancreat Dis Int. 2008;7:406–411. 18693177

[138] You Q, Cheng L, Kedl RM, Ju C. Mechanism of T cell tolerance induction by murine hepatic Kupffer cells. Hepatology. 2008;48:978–990. doi: 10.1002/hep.22395 18712788 PMC2600585

[139] Song J, Ge Z, Yang X, Luo Q, Wang C, You H, et al. Hepatic stellate cells activated by acidic tumor microenvironment promote the metastasis of hepatocellular carcinoma via osteopontin. Cancer Lett. 2015; 356:713–720. doi: 10.1016/j.canlet.2014.10.021 25449435

[140] Novikova MV, Khromova NV, Kopnin PB. Components of the hepatocellular carcinoma microenvironment and their role in tumor progression. Biochemistry (Mosc). 2017;82(8):861–873. doi: 10.1134/S0006297917080016 28941454

[141] Patten DA, Shepherd EL, Weston CJ, Shetty S. Novel targets in the immune microenvironment of the hepatic sinusoids for treating liver diseases. Semin Liver Dis 2019; 39: 111–123 doi: 10.1055/s-0039-1678727 30912097

[142] Omenetti A, Porrello A, Jung Y, Yang L, Popov Y, Choi SS, et al. Hedgehog signaling regulates epithelial-mesenchymal transition during biliary fibrosis in rodents and humans. J Clin Invest. 2008;118:3331–3342. doi: 10.1172/JCI35875 18802480 PMC2542850

[143] Helal M, Yan C, Gong Z. Stimulation of hepatocarcinogenesis by activated cholangiocytes via II17a/f1 pathway in kras transgenic zebrafish model. Sci Rep 2021; 11:1372 33446803 10.1038/s41598-020-80621-6 PMC7809472

