# Supplementary material for: Down-regulation of hepatic expression of GHR/STAT5/IGF-1 signaling pathway fosters development and aggressiveness of HCV-related hepatocellular carcinoma: Crosstalk with Snail-1 and type 2 transforming growth factor-beta receptor
Source: PLoS One. 2022 Nov 14;17(11):e0277266. doi: 10.1371/journal.pone.0277266 (PMC9662744; doi:10.1371/journal.pone.0277266)
Supplement: Attachment — Submitted filename: Dear Dr.docx [file pone.0277266.s001.docx]

**Dear Dr. Gianfranco D. Alpini**

**Academic Editor**

**Plos One**

I hope this email finds you well. First and foremost, I sincerely appreciate your positive outlook and support. The reply for the reviewer #2 comment is:

According to his valuable advice, the images are presented at 20X magnification power with a 200X zoom in box (c.f., Figure 3). I hope the images resolution will be accepted.

Kind regards,

Prof. Dr. Mona A. Abu El-Makarem

ORCID: 0000-0001-9202-4373

July 24, 2022
