# Supplementary material for: Down-regulation of hepatic expression of GHR/STAT5/IGF-1 signaling pathway fosters development and aggressiveness of HCV-related hepatocellular carcinoma: Crosstalk with Snail-1 and type 2 transforming growth factor-beta receptor
Source: PLoS One. 2022 Nov 14;17(11):e0277266. doi: 10.1371/journal.pone.0277266 (PMC9662744; doi:10.1371/journal.pone.0277266)
Supplement: Attachment — Submitted filename: Dear Dr.docx [file pone.0277266.s002.docx]

**Dear Dr. Gianfranco D. Alpini**

**Academic Editor**

**Plos One**

I trust this email finds you well. The reply for journal requirements is:

I would like to assure you that the reference list becomes complete, correct and does not contain any retracted reference.

Note:

In references no.: 4, 14, 52, 53, 54, 64, 79, 84, 102, 108, 123, 125, 128, 131, 136 and 144, only the front page numbering is available according to the publication guidelines.

The reply for the reviewer #2 comment is:

I sincerely apologize for any inconvenience caused. All slides have been retaken to get clearer pictures, and this is the most possible for the efficiency of the devices available in our institute.

Kind regards,

Prof. Dr. Mona A. Abu El-Makarem

ORCID: 0000-0001-9202-4373

26-9- 2022
